# Systematic Studies on the Anti-SARS-CoV-2 Mechanisms of Tea Polyphenol-Related Natural Products

**DOI:** 10.1021/acsomega.4c02392

**Published:** 2024-05-17

**Authors:** Chen-Wei Li, Tai-Ling Chao, Chin-Lan Lai, Cheng-Chin Lin, Max Yu-Chen Pan, Chieh-Ling Cheng, Chih-Jung Kuo, Lily Hui-Ching Wang, Sui-Yuan Chang, Po-Huang Liang

**Affiliations:** † Institute of Biological Chemistry, Academia Sinica, Taipei 11529, Taiwan; ‡ Institute of Biochemical Sciences, National Taiwan University, Taipei 10617, Taiwan; § Department of Clinical Laboratory Sciences and Medical Biotechnology, National Taiwan University, Taipei 10048, Taiwan; ∥ Institute of Molecular and Cellular Biology, National Tsing Hua University, Hsinchu 30013, Taiwan; ⊥ Department of Veterinary Medicine, National Chung Hsing University, Taichung 40227, Taiwan; # Department of Laboratory Medicine, National Taiwan University Hospital, Taipei 10002, Taiwan

## Abstract

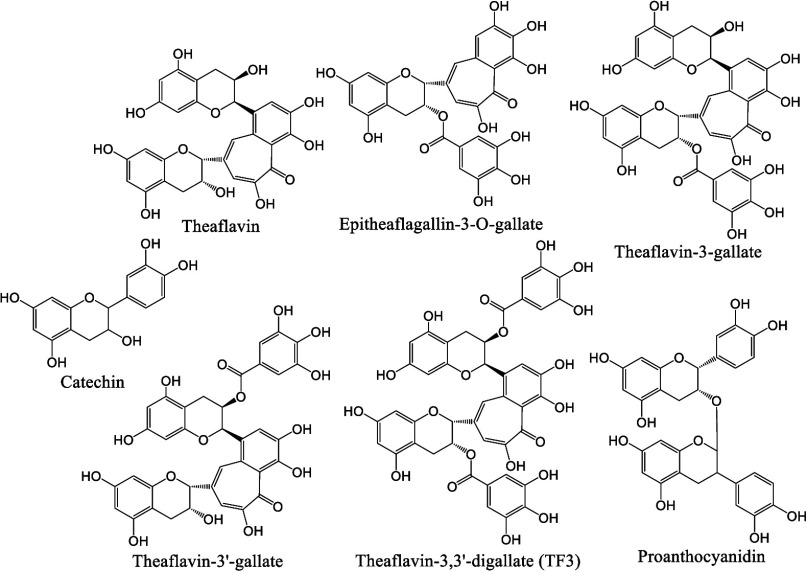

The causative pathogen of COVID-19, severe acute respiratory syndrome-coronavirus-2 (SARS-CoV-2), utilizes the receptor-binding domain (RBD) of the spike protein to bind to human receptor angiotensin-converting enzyme 2 (ACE2). Further cleavage of spike by human proteases furin, TMPRSS2, and/or cathepsin L facilitates viral entry into the host cells for replication, where the maturation of polyproteins by 3C-like protease (3CL^pro^) and papain-like protease (PL^pro^) yields functional nonstructural proteins (NSPs) such as RNA-dependent RNA polymerase (RdRp) to synthesize mRNA of structural proteins. By testing the tea polyphenol-related natural products through various assays, we found that the active antivirals prevented SARS-CoV-2 entry by blocking the RBD/ACE2 interaction and inhibiting the relevant human proteases, although some also inhibited the viral enzymes essential for replication. Due to their multitargeting properties, these compounds were often misinterpreted for their antiviral mechanisms. In this study, we provide a systematic protocol to check and clarify their anti-SARS-CoV-2 mechanisms, which should be applicable for all of the antivirals.

## 1 Introduction

Since its emergence in 2019 December, the coronavirus disease 2019 (COVID-19) has infected approximately 0.7 billion people and resulted in 6.9 million deaths. The pandemic continues with a case fatality rate of ∼1%.^1,2^ The causative agent of COVID-19 shares genomic homology with the severe acute respiratory syndrome-coronavirus (SARS-CoV) that caused an outbreak in 2002–2003^3,4^ and was thus named SARS-CoV-2 by the World Health Organization (WHO). These human coronaviruses (CoVs) belong to the β genera of the CoV subfamily, which consists of single-stranded positive-sense RNA viruses. The viral infection is initiated by binding of the receptor-binding domain (RBD) of spike on SARS-CoV and SARS-CoV-2 to human receptor angiotensin-converting enzyme 2 (ACE2). After virus attachment, spike is initially cleaved by the human transmembrane protease furin at the S1/S2 site, dividing it into S1 and S2 fragments, followed by cleavage at the S2 site through human transmembrane serine protease 2 (TMPRSS2) to facilitate membrane fusion for viral RNA entry.^5^ Alternatively, the virus may undergo endocytosis mediated by ACE2 to form an endosome. Within the endosome, the spike is cleaved by cathepsin L at the S1/S2 site to facilitate membrane fusion and the subsequent release of the viral RNA into the cytosol.^6^

After virus entry, the positive-sense viral RNA is translated into two viral polyproteins, pp1a and pp1ab, by the host cellular machinery. These polyproteins are then cleaved by the virus-encoded papain-like protease (PL^pro^) and 3C-like protease (3CL^pro^), which are embedded within the polyproteins and are self-cleaved for maturation, to yield 16 mature nonstructural proteins (NSPs).^7^ While 3CL^pro^ primarily facilitates the maturation of NSPs, PL^pro^ exhibits multifaceted activities, including polyprotein cleavage, removal of ubiquitin, and deISGylation of interferon (IFN)-stimulated gene product 15 (ISG15) to antagonize host immunity.^8,9^ Subsequently, NSP12 forms a complex with two accessories NSP7 and NSP8, possessing RNA-dependent RNA polymerase (RdRp) activity.^10,11^ Additionally, RdRp collaborates with helicase (NSP13)^12^ as well as several RNA-processing enzymes such as NSP14, a bifunctional enzyme with 30-to-50 exoribonuclease (ExoN) and N7-methyltransferase activities,^13^ to form the replication–transcription complex, which could further synthesize mRNA from RNA(+) and generate four structural proteins, spike (S), nucleocapsid (N), membrane (M), and envelope (E) proteins for assembly of new virus particles.

**Figure 1:**
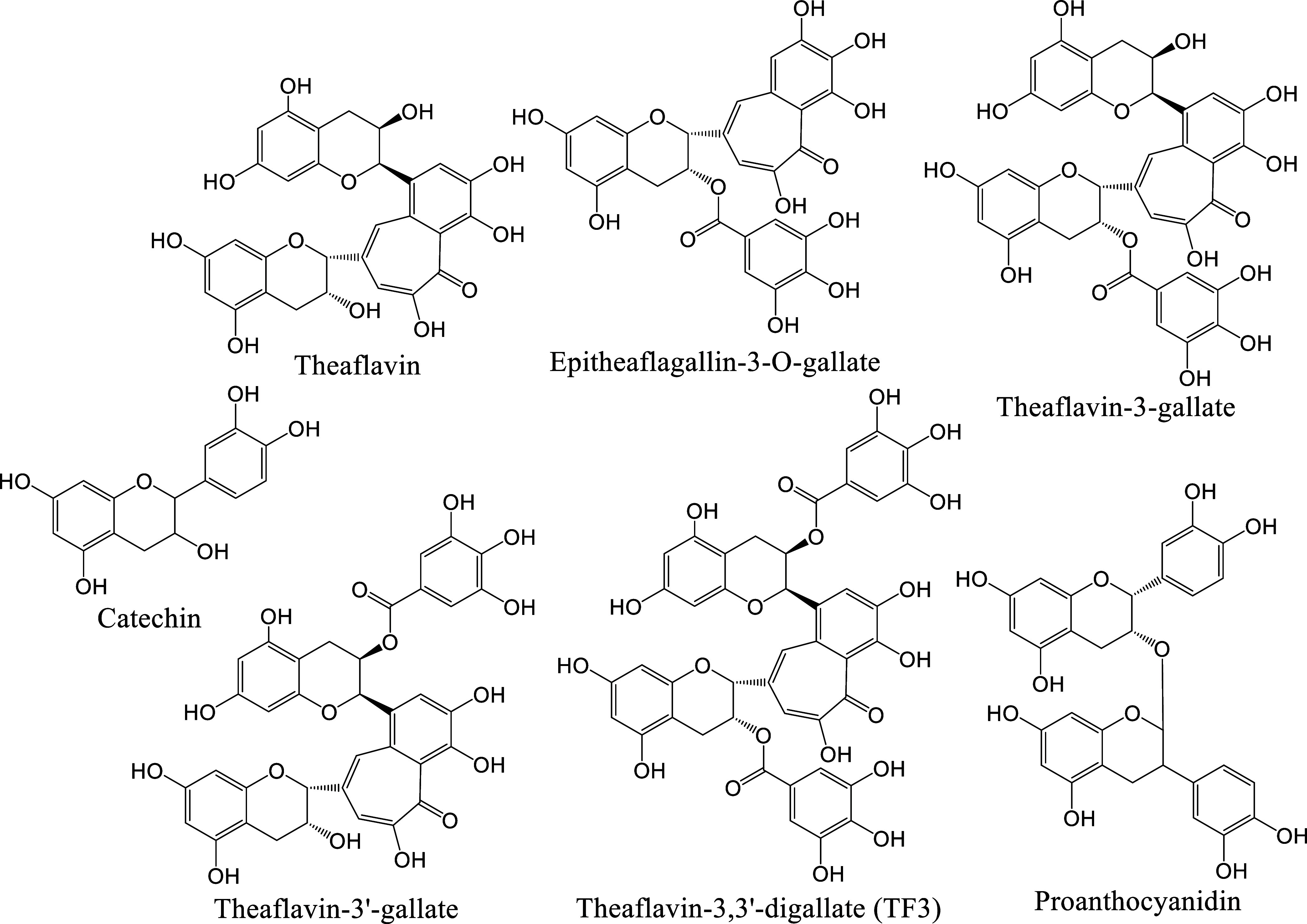
Chemical structures of the natural products evaluated in this study. The tea polyphenol-related natural products tested include catechin, theaflavin, epitheaflagallin-3-*O*-gallate, theaflavin-3-gallate, theaflavin-3′-gallate, TF3, and proanthocyanidin.

Essential for virus entry and replication, the RBD/ACE2 interaction, TMPRSS2/furin/cathepsin L, 3CL^pro^/PL^pro^, RdRp, and others represent promising targets for anti-SARS-CoV-2 against COVID-19.^14−16^ Among the food and drug administration (FDA)-approved anti-COVID-19 drugs, antibodies such as bebtelovimab hinder viral attachment by binding to the RBD to inhibit its interaction with ACE2.^17^ Remdesivir and molnupiravir inhibit RdRp,^18−20^ while Paxlovid (active ingredient: nirmatrelvir) inhibits 3CL^pro^.^21^ With the emergence of drug resistance,^22−25^ more drug candidates are desired. Natural products, known to have generally mild adverse effects, have been previously suggested for the treatment and/or prevention of SARS disease^26−29^ and COVID-19.^30−33^ However, the antiviral mechanisms of certain effective natural products, such as tea polyphenols, remain incompletely understood due to their multiple-targeting properties. These natural products have been assessed through limited enzymatic assays and pseudovirus entry assays and/or predicted as inhibitors via computer modeling (refer to the Section 4) to be classified as anti-SARS-CoV-2 agents by inhibiting the targets assayed. It is now recognized that blocking the interaction between RBD and ACE2 as well as inhibiting enzyme activities of TMPRSS2, furin, and/or cathepsin L could block virus entry, whereas targeting viral enzymes like PL^pro^, 3CL^pro^, and/or RdRp could antagonize virus replication. Whether the antivirals inhibit virus entry or replication could be distinguished by assessing their effectiveness through pre-entry and postentry treatments. In this study, we systematically investigated the inhibitory effects of a series of tea polyphenol-related natural products (chemical structures shown in Figure 1) on various targets, including RBD/ACE2 interaction, TMPRSS2, furin, cathepsin L, 3CL^pro^, PL^pro^, and RdRp, and elucidated their antiviral mechanisms through entry or postentry treatment, to clarify their antiviral mechanisms.

## 2 Experimental Methods

### 2.1 Materials

All of the natural products used herein were purchased from MedChemExpress (NJ), with the exception of epitheaflagallin-3-*O*-gallate obtained from GlpBio (CA), theaflavin-3-gallate from Cayman Chemical (MI), and TF3 from ChromaDex, Inc. (CA). All of the chemical reagents employed were of the highest grade.

### 2.2 Expression and Purification of Recombinant TMPRSS2

The recombinant TMPRSS2 ectodomain (residues 109–492), excluding the transmembrane domain, was expressed using the ExpiSf Baculovirus Expression System (Thermo Fisher, catalog no. A38841, A39111, A39112) as reported previously.^34,35^ Briefly, the *Escherichia coli* DH10Bac cells were transformed with the TMPRSS2 plasmid to generate viral bacmid DNA, which was subsequently used to transfect ExpiSf cells for the production of recombinant baculovirus particles. These viral particles were then amplified from the P0 to P1 viral stocks. Recombinant P1 viruses were used to infect ExpiSf9 insect cells in ExpiSf CD medium. After 4 days of infection with cell viability dropping to 40–50%, the cell culture containing the secreted His-tagged TMPRSS2 was loaded onto a HisTrap column (Cytiva) to capture the target protein. The HisTrap column was washed with phosphate-buffered saline (PBS) buffer containing up to 25 mM imidazole and then eluted with the buffer containing up to 250 mM imidazole. The partially purified protein was then activated by the addition of enterokinase (NEB) and subjected to further purification using a Superdex 75 10/300 GL column (GE Healthcare).

### 2.3 TMPRSS2 Inhibition Assays

TMPRSS2 activity was assayed using a fluorogenic substrate Boc-Gln-Ala-Arg-AMC (Bachem, catalog no. 4017019.0025, Switzerland) as reported previously.^34,35^ A microplate reader (BioTek Synergy H1) was used to measure the fluorescence increases at excitation and emission wavelengths of 355/460 nm. The assay was conducted in the presence of 1.3 nM TMPRSS2, 10 μM substrate, and various concentrations of inhibitors in a buffer of 20 mM tris–HCl (pH 7.4) containing 1% dimethyl sulfoxide (DMSO) from the stock solutions of inhibitors. The concentration-dependent inhibition curves of TMPRSS2 were fitted with the equation A(*I*) = A(0) x {1–[*I*/(*I* + IC_50_)]} using GraphPad Prism (v.9.4.0) software to determine the IC_50_ values. Here, A(*I*) represents the enzyme activity at a given inhibitor concentration *I*, while A(0) denotes the enzyme activity in the absence of inhibitor, and *I* represents the inhibitor concentration. All of the measurements were triplicated to calculate the averaged IC_50_ values and the standard deviations.

### 2.4 Furin Inhibition Measurements

Following the protocol as described in ref (35) a commercial Furin Protease Assay Kit (BPS Bioscience, catalog#78040) was employed to assay furin activities. It was conducted in 20 μL reaction mixtures on a 384-well plate (PerkinElmer) comprising 0.25 ng/μL furin, 2 μM substrate, and different concentrations of inhibitors in the assay buffer with 1% DMSO from the stock solutions of compounds. Fluorescence intensity was monitored at excitation/emission wavelengths of 380/460 nm. The concentration-dependent inhibition curves of furin were fitted with the equation A(*I*) = A(0) x {1–[*I*/(*I* + IC_50_)]} using GraphPad Prism (v.9.4.0). All of the measurements were performed in triplicate to determine the averaged IC_50_ values and the standard deviations.

### 2.5 Cathepsin L IC_50_ Measurements

Cathepsin L activity was determined using a commercial Cathepsin L Protease Assay Kit (Abcam, U.K.) in 20 μL reaction mixtures on a 384-well plate (PerkinElmer). Different concentrations of each inhibitor were added to the assay buffer supplied with dithiothreitol (DTT) in the kit, also containing 1% DMSO from the stock solutions of inhibitors. The fluorescence intensity was monitored for 30 min at excitation and emission wavelengths of 380/460 nm. The concentration-dependent inhibition curves of cathepsin L were fitted with the equation A(*I*) = A(0) x {1–[*I*/(*I* + IC_50_)]} using GraphPad Prism (v.9.4.0). The measurements were performed in triplicate to generate the averaged IC_50_ values and the standard deviations.

### 2.6 RBD/ACE2 Cell-Based Binding Assay

The inhibition of RBD/ACE2 binding by the natural products was evaluated by using the NanoBiT technology commercial kit from Promega (WI). A stable cell line expressing SmBiT-tagged human ACE2 on HeLa cells was established, and the recombinant RBD-LgBiT protein (amino acids 330–521 of spike) was produced.^35,36^ To monitor the interaction between RBD and ACE2, SmBiT-ACE2-expressing cells were seeded onto a white 96-well plate at a density of 1 × 10^4^ cells per well (in triplicate). For each binding assay, cells were washed once with PBS and pretreated with 50 μL of the indicated compounds per well for 10 min. Next, a 50 μL reaction mixture containing 10 ng of RBD-LgBiT, 0.5 μL of Nano-Glo luciferase assay substrate, and 9.5 μL of luciferase assay diluent (Promega) was added into each well. The luminescence signal was recorded every 2 min continuously for 1 h using a microplate reader (BioTek Synergy HTX, VT) at 37 °C with a time-lapsed kinetics program. To calculate RBD inhibition by all agents, luminescence data from the time point showing the highest signal in the negative control sample was selected for downstream calculation. Inhibition (%) = [1-(luminescence signal of test sample)/(luminescence signal of negative control sample)] × 100. The compound concentration required to inhibit 50% of the interaction is defined as IC_50_. All of the measurements were performed in triplicate to determine the averaged IC_50_ values and the standard deviations.

### 2.7 3CL^pro^ Inhibition Measurements

Recombinant SARS-CoV-2 3CL^pro^ was prepared as previously reported.^37^ The purified tag-free 3CL^pro^ was dialyzed against a buffer containing tris–HCl (pH 7.5), 120 mM NaCl, 0.1 mM ethylenediaminetetraacetic acid (EDTA), and 2 mM DTT and stored at −70 °C until use. 3CL^pro^ activity was monitored using a fluorogenic peptide Dabcyl-KTSAVLQSGFRKME-Edans purchased from Yuan Yu Ltd. (Taiwan). This peptide contained a fluorescence quenching pair, and the fluorescence increased when the peptide was cleaved by the protease. Fluorescence resulting from the cleavage of the fluorogenic peptide by 3CL^pro^ was measured over time at 538 nm with excitation at 355 nm using a fluorescence plate reader. IC_50_ values of the active compounds were measured in reaction mixtures containing 35 nM 3CL^pro^ and 6 μM fluorogenic substrate in a buffer of 20 mM Bis-tris (pH 7.0) in the absence and presence of various concentrations of inhibitors. No additional reducing agent was added since the 3CL^pro^ storage buffer already contained 2 mM DTT, which should be present in the assay buffer as well. It is important to note that previous studies have demonstrated that in the absence of DTT, compounds like ebselen, disulfiram, carmofur, PX-12, tideglusib, and shikonin exhibit nonspecific inhibition not only against 3CL^pro^ but also against a panel of viral cysteine proteases, including SARS-CoV-2 PL^pro^, as well as 2A^pro^ and 3C^pro^ from enteroviruses A71 and D68.^38^ All of the measurements were triplicated to yield the averaged IC_50_ and standard deviations.

### 2.8 PL^pro^ IC_50_ Measurements

Recombinant SARS-CoV-2 PL^pro^ was prepared as previously reported.^37^ The purified PL^pro^ was dialyzed against a buffer containing tris–HCl (pH 7.5), 120 mM NaCl, 0.1 mM EDTA, and 2 mM DTT and stored at −70 °C until use. For the determination of IC_50_ against proteolytic, deubiquitinating, and deISGylation activities of PL^pro^, the following fluorogenic substrates were utilized: Z-Arg-Leu-Arg-Gly-Gly-AMC (Cat. #79997, Bachem Bioscience, Switzerland) for proteolytic activity, Ub-AMC (Cat. #U550-050, Boston Biochem) for deubiquitinating activity, and ISG15-AMC (Cat. #U553-050, Boston Biochem) for deISGylation activity. The initial rates of the reactions involving 75 nM PL^pro^ and 10 μM fluorogenic substrate were measured in a buffer of 20 mM HEPES (pH 7.5), in both the absence and presence of various concentrations of inhibitors. The measurements were taken at 460 nm upon excitation at 355 nm. No additional reducing agent was added as 2 mM DTT was already present in the PL^pro^ storage buffer and should be present in the assay buffer as well. The initial rates of the inhibited reactions were plotted against different inhibitor concentrations to determine the IC_50_ value by fitting with the following equation: A(*I*) = A(0) × {1-[*I*/(*I* + IC_50_)]}. All of the measurements were performed in triplicate to determine the averaged IC_50_ values and the standard deviations.

### 2.9 Preparation and Assay of SARS-CoV-2 RdRp

The fusion protein nsp7–nsp8 (nsp7L8) was generated by inserting a GSGSGS linker sequence between the nsp7 and nsp8 coding sequences.^11^ The nsp7L8, nsp8, and nsp12 were produced and purified independently. The procedure involved using both *E. coli* for expressing nsp7L8 and baculovirus-infected insect cells for expressing nsp12.^39,40^ Subsequently, nsp12, nsp7L8, and nsp8 were combined at a molar ratio of 1:3:3 and preincubated on ice for 10 min to facilitate the formation of an active complex following a reported protocol.^41,42^ In vitro RdRp activity was monitored using a fluorescence plate reader (Synergy H1 Hybrid multimode Reader, BioTek) with a commercial kit (ProFoldin, Huson, MA). The initial rates of the inhibited reactions were plotted against different inhibitor concentrations to determine the IC_50_ value by fitting with the equation: A(*I*) = A(0) × {1–[*I*/(*I* + IC_50_)]}. All of the measurements were performed in triplicate to determine the averaged IC_50_ values and the standard deviations.

### 2.10 Antiviral EC_50_ Measurements

Vero E6 cells were seeded onto a 24-well culture plate in Dulbecco’s modified Eagle medium (DMEM) with 10% fetal bovine serum (FBS) and antibiotics 1 day before infection. For entry treatment, SARS-CoV-2 Delta virus (NTU92) or Omicron BA.5 (NTU280) at 50–100 plaque-forming units (pfu) was preincubated with test compounds for 1 h at 37 °C before being added to cells. Then, the mixture was incubated for an additional hour. After the removal of virus inoculum, the cells were washed once with phosphate-buffered saline (PBS) and overlaid with 1 mL of overlay medium containing 1% methylcellulose, with or without test compounds for postentry treatment or entry treatment, respectively. After a 5 day incubation at 37 °C, the cells were fixed with 10% formalin overnight. Following the removal of the overlay media, the cells were stained with 0.5% crystal violet, and the plaques were counted. The percentage of inhibition was calculated as [1–(*V*_D_/*V*_C_)] × 100%, where *V*_D_ and *V*_C_ represent the virus titer in the presence and absence of test compounds, respectively. The minimal concentrations of compounds required to reduce 50% of plaque numbers (EC_50_) were calculated by regression analysis of the dose–response curves generated from plaque assays. For each compound, the measurements were repeated at least 3 times to obtain the averaged EC_50_ values and standard deviation. To ascertain the stages at which the compounds exerted their antiviral effects, the compounds were added before infection, during infection, and/or after infection, as described in the Section 3.

### 2.11 Cytotoxicity CC_50_ Measurements

Cytotoxicity of the compounds was determined using the acid phosphatase (ACP) assay. Vero E6 cells were seeded onto a 96-well culture plate at a concentration of 2 × 10^4^ cells per well. Next day, the medium was removed, and each well was washed once with PBS before adding DMEM containing 2% FBS and different concentrations of test compounds. After 3 days of incubation at 37 °C, the medium was removed, and each well was washed once with PBS. Next, a buffer containing 0.1 M sodium acetate (pH = 5.0), 0.1% Triton X-100, and 5 mM *p*-nitrophenyl phosphate was added. After incubating at 37 °C for 30 min, 1 N NaOH was added to stop the reaction. The absorbance was measured using an ELISA reader (VERSAmax, Molecular Devices, Sunnyvale, CA) at a wavelength of 405 nm. The percentage of cell viability was calculated by using the following formula: Viability % = [ (*A*_t_/*A*_s_) × 100]%, where At and As represent the absorbance of the test compound and the solvent control, respectively. The 50% cytotoxicity concentration (CC_50_) was determined by nonlinear regression analysis. For each compound, the measurements were repeated at least 3 times to determine the averaged CC_50_ values and standard deviation.

### 2.12 Molecular Docking

The molecular docking analysis was conducted utilizing the Discovery Studio (DS) 2022 software to forecast the interaction between natural products and TMPRSS2, furin, and cathepsin L. The three-dimensional (3D) structures of TMPRSS2 (PDB: 7MEQ), furin (PDB: 4RYD), and cathepsin L (PDB: 5MQY) were retrieved from the RCSB Protein Data Bank (PDB, https://www.rcsb.org/), with the elimination of all water molecules and bound ligands. The binding sites were prepared by extracting the binding cavity surrounding nafamostat, a recognized inhibitor of TMPRSS2, with 12 Å radius. Similarly, the binding cavity encompassing para-guanidinomethyl-Phac-R-Tle-R-Amba, a known furin inhibitor, was extracted with the same radius as that for furin. The binding cavity of cathepsin L was determined analogous to that of TMPRSS2 and furin. The natural products were prepared using the built-in function in the DS software for ligand preparation. Prior to docking, all proteins and ligands were optimized to ensure stable conformations.

## 3 Results

### 3.1 IC_50_ of the Compounds for Inhibiting Cathepsin L, Furin, and/or TMPRSS2

**Figure 2:**
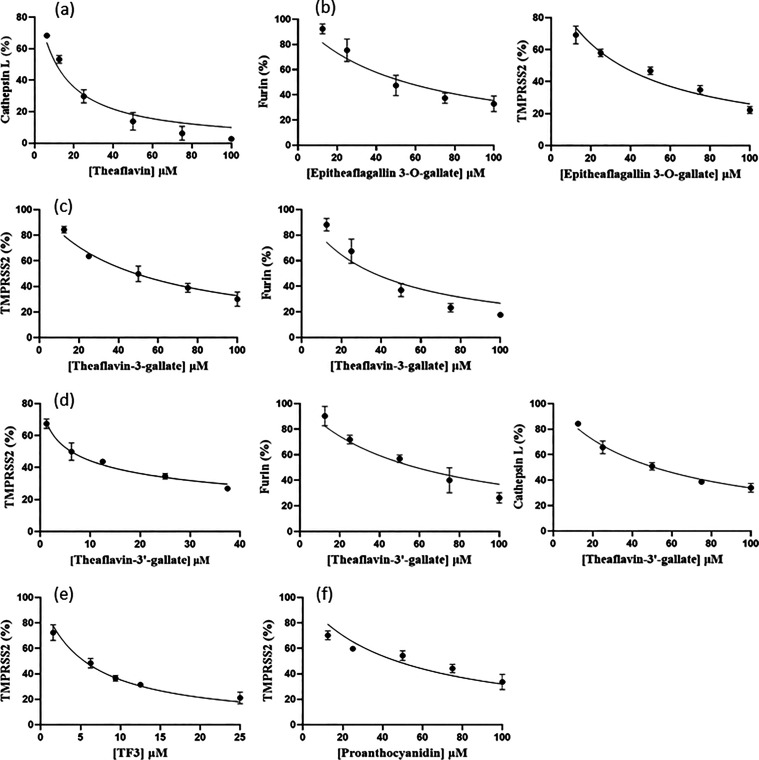
IC_50_ measurements of compounds against cathepsin L, furin, and/or TMPRSS2. (a) Theaflavin inhibited cathepsin L with IC_50_ values of 11.1 ± 1.1 μM. (b) Epitheaflagallin-3-*O*-gallate inhibited furin and TMPRSS2 with IC_50_ values of 55.0 ± 6.5 and 35.3 ± 2.7 μM, as shown in the left and right panels, respectively. (c) Theaflavin-3-gallate inhibited TMPRSS2 and furin with IC_50_ values of 48.7 ± 3.0 and 36.7 ± 5.7 μM, as shown in the left and right panels, respectively. (d) Theaflavin-3′-gallate inhibited TMPRSS2, furin, and cathepsin L with IC_50_ values of 6.2 ± 0.6, 58.5 ± 6.4, and 51.1 ± 2.2 μM, as shown in the left, middle, and right panels, respectively. (e, f) TF3 and proanthocyanidin inhibited TMPRSS2 showing IC_50_ values of 5.5 ± 0.4 and 47.0 ± 4.6 μM, respectively. All of the measurements were performed in triplicate to yield the averaged IC_50_ values and the standard deviations.

To systematically examine the anti-SARS-CoV-2 mechanisms, seven related polyphenols were selected. Their structures are shown in the order of smaller to larger sizes from top-left to bottom-right, including catechin, theaflavin, epitheaflagallin-3-*O*-gallate, theaflavin-3-gallate, theaflavin-3′-gallate, theaflavin-3,3′-digallate (TF3), and proanthocyanidin (Figure 1). Catechin is a flavanol; theaflavin is a catechin fused with its analogue containing a 2-hydroxy-2,4,6-cycloheptatrien-1-one ring; theaflavin-3-gallate, theaflavin-3′-gallate, and TF3 are theaflavins with gallate substitutions on either side or both sides, respectively; epitheaflagallin-3-*O*-gallate resembles theaflavin-3-gallate but lacks a chromane group; and proanthocyanidin contains two catechin molecules linked by an oxygen atom. In our study, the inhibitory effects of these natural products containing catechin and analogues on TMPRSS2, furin, and/or cathepsin L, the human proteases involved in SARS-CoV-2 entry, were investigated using established assays.^34,35^ We observed that the small theaflavin inhibited cathepsin L with an IC_50_ of 11.1 ± 1.1 μM (Figure 2a). Additionally, the larger epitheaflagallin-3-*O*-gallate inhibited furin and TMPRSS2 with IC_50_ values of 55.0 ± 6.5 and 35.3 ± 2.7 μM, respectively (Figure 2b left and right panels, respectively). Furthermore, theaflavin-3-gallate inhibited TMPRSS2 and furin with IC_50_ values of 48.7 ± 3.0 and 36.7 ± 5.7 μM, respectively (Figure 2c left and right panels, respectively). Theaflavin-3′-gallate inhibited TMPRSS2, furin, and cathepsin L with IC_50_ values of 6.2 ± 0.6, 58.5 ± 6.4, and 51.1 ± 2.2 μM, respectively (Figure 2d left, middle, and right panels, respectively), and the even larger TF3 and proanthocyanidin inhibited TMPRSS2 with IC_50_ values of 5.5 ± 0.4 and 47.0 ± 4.6 μM (Figure 2e,f), respectively. These IC_50_ values are summarized in Table 1. The assays revealed distinct inhibitor profiles: the smaller theaflavin primarily inhibited cathepsin L, while the middle-sized epitheaflagallin-3-*O*-gallate and theaflavin-3- gallate showed weak inhibition against furin and TMPRSS2. Theaflavin-3′-gallate inhibited all three proteases and was particularly potent on TMPRSS2. The larger TF3 and proanthocyanidin selectively inhibited TMPRSS2, with TF3 demonstrating stronger potency. This structure–activity relationship suggests an enlargement of binding cavities from cathepsin L and furin to TMPRSS2 to accommodate increasing sizes of polyphenols, which is supported by simulated binding modes as shown later.

**Table 1:** IC_50_, EC_50_, and CC_50_ of the Natural Products with Related Structures

	IC_50_ (μM)								
natural products	cathepsin L	furin	TMPRSS2	RBD/ACE2	3CL^pro^	PL^pro^	RdRp	EC_50_ (μM) δ/ο BA.5	CC_50_ (μM)
catechin	>100	>100	>100	>100	>100	18.7 ± 3.5	>100	>10/>10	106.7
						24.6 ± 3.3			
						17.8 ± 2.2			
theaflavin	11.1 ± 1.1	>100	>100	12.3 ± 1.1	16.1 ± 2.5	9.9 ± 0.9	40.2 ± 2.3	7.4 ± 0.1/2.2 ± 0.1	66.4 ± 3.2
						10.6 ± 0.9			
						11.2 ± 1.3			
epitheaflagallin-3-*O*-gallate	>100	55.0 ± 6.5	35.3 ± 2.7	13.6 ± 1.1	31.2 ± 2.6	>100	>100	0.5 ± 0.1/0.6 ± 0.3	109.2 ± 45.3
theaflavin-3-gallate	>100	36.7 ± 5.7	48.7 ± 3.0	9.9 ± 1.1	8.8 ± 0.8	>100	>100	0.4 ± 0.0/0.4 ± 0.0	>100
theaflavin-3′-gallate	51.1 ± 2.2	58.5 ± 6.4	6.2 ± 0.6	3.0 ± 1.1	6.7 ± 0.8	>100	23.2 ± 0.4	0.8 ± 0.1/0.4 ± 0.1	69.9 ± 12.9
theaflavin-3,3′-digallate (TF3)	>100	>100	5.5 ± 0.4		8.5 ± 0.7	4.7 ± 0.5	2.3 ± 0.6	0.2 ± 0.0/0.2 ± 0.1	>100
				8.7 ± 1.2		5.5 ± 0.6			
						6.3 ± 0.8			
proanthocyanidin	>100	>100	47.0 ± 4.6	12.4 ± 1.1	18.4 ± 1.1	2.4 ± 0.3	23.4 ± 1.3	0.5 ± 0.1/0.2 ± 0.2	>100
						3.3 ± 0.4			
						7.3 ± 0.6			

### 3.2 Cell-Based Assay of the Compounds in Inhibiting the RBD/ACE2 Interaction

**Figure 3:**
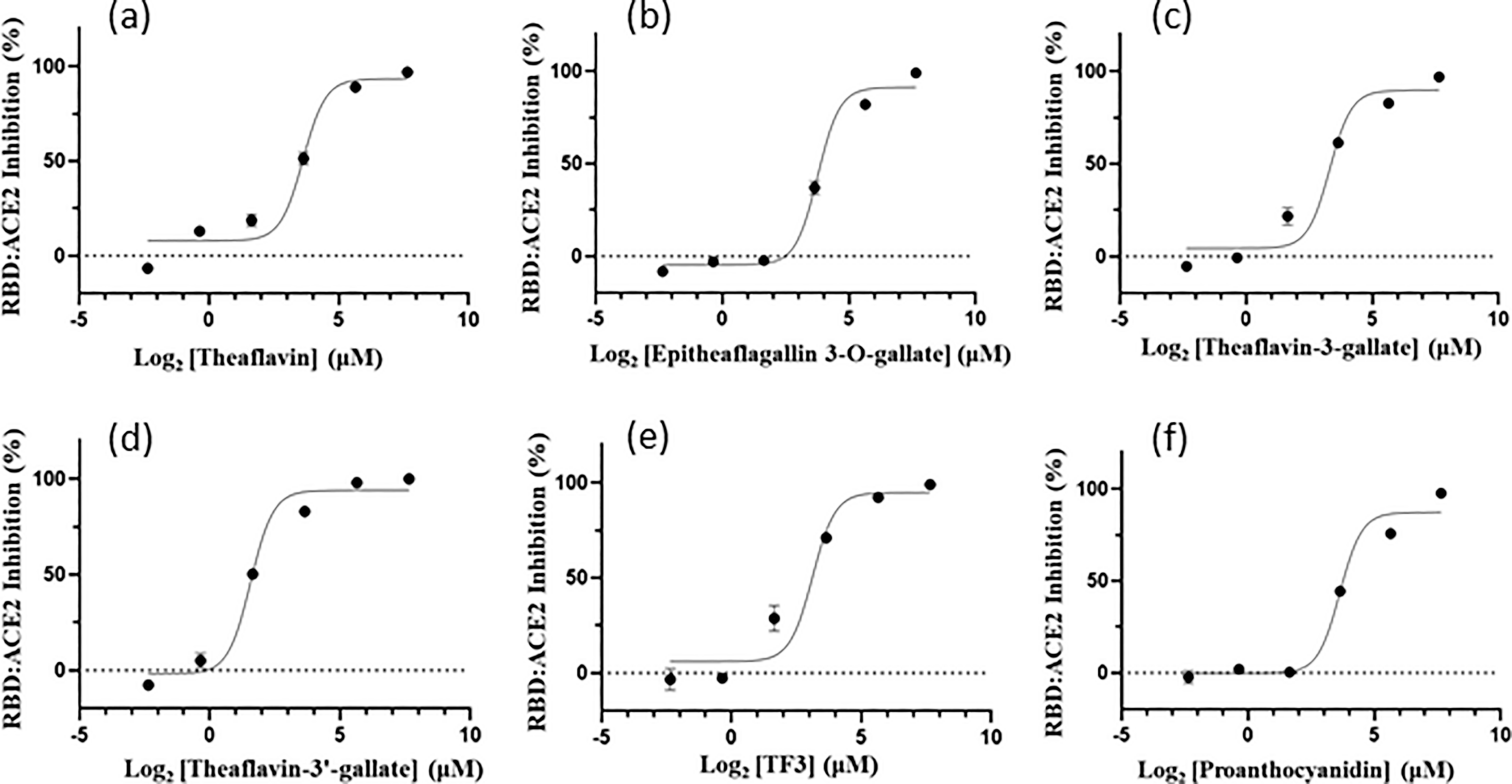
Interference of compounds with the RBD/ACE2 interaction. The indicated compounds were preincubated with the recombinant RBD proteins before subjected to the RBD/ACE2 binding assay. (a–f) Percentages of RBD/ACE2 binding inhibition with increasing compound concentrations were measured with RBD from Delta SARS-CoV-2 to yield IC_50_ values of 12.3 ± 1.1, 13.6 ± 1.1, 9.9 ± 1.1, 3.0 ± 1.1, 8.7 ± 1.2, and 12.4 ± 1.1 μM for theaflavin, epitheaflagallin-3-*O*-gallate, theaflavin-3-gallate, theaflavin-3′-gallate, TF3, and proanthocyanidin, respectively. All measurements were performed in triplicate to yield the averaged IC_50_ values and standard deviations.

The cell-based measurements of the inhibitory effects of natural products against RBD/ACE2 were determined using a commercial kit of NanoBiT technology (Promega, WI) as previously described.^35,36^ As shown in Figure 3a–f, a dose-dependent inhibition of the binding between the recombinant RBD of the Delta strain spike and ACE2 by theaflavin, epitheaflagallin-3-*O*-gallate, theaflavin-3-gallate, theaflavin-3′-gallate, TF3, and proanthocyanidin was observed, yielding IC_50_ values of 12.3 ± 1.1, 13.6 ± 1.1, 9.9 ± 1.1, 3.0 ± 1.1, 8.7 ± 1.2, and 12.4 ± 1.1 μM, respectively. These IC_50_ values are summarized in Table 1.

### 3.3 Assay of the Compounds for Inhibiting Enzyme Activities of 3CL^pro^ and PL^pro^

**Figure 4:**
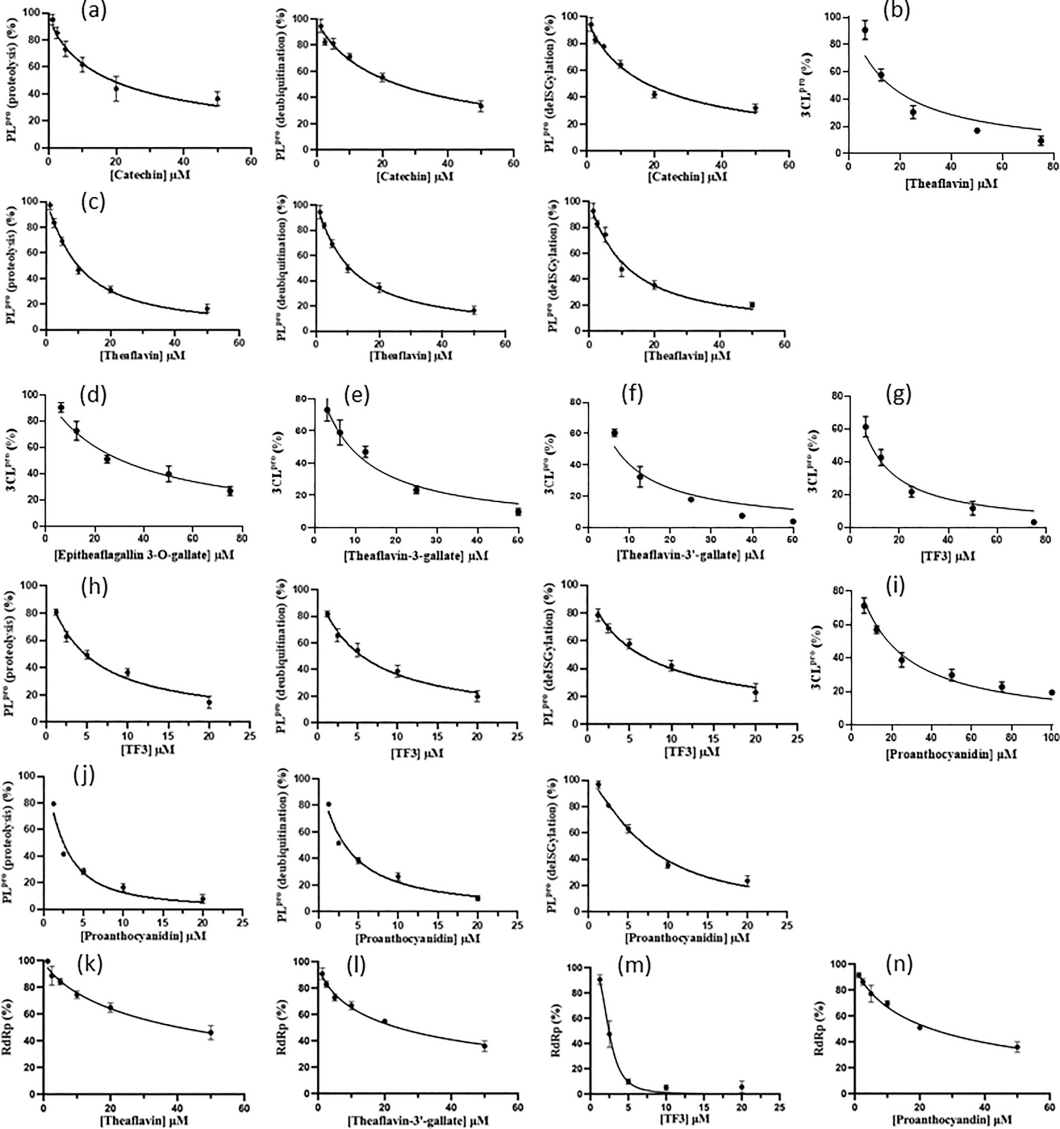
IC_50_ measurements of the compounds against 3CL^pro^, PL^pro^, and RdRp. (a) Catechin inhibited three activities of PL^pro^ with IC_50_ values of 18.7 ± 3.5, 24.6 ± 3.3, and 17.8 ± 2.2 μM, respectively, based on the inhibitor concentration-dependent curves shown in the left, middle, and right panels, respectively. (b, c) Theaflavin inhibited 3CL^pro^ and the three activities of PL^pro^ with IC_50_ values of 16.1 ± 2.5, 9.9 ± 0.9, 10.6 ± 0.9, and 11.2 ± 1.3 μM, respectively, based on the inhibitor concentration-dependent curves shown in left, middle, and right panels, respectively. (d–f) Epitheaflagallin-3-*O*-gallate, theaflavin-3-gallate, theaflavin-3′-gallate inhibited 3CL^pro^ with IC_50_ values of 31.2 ± 2.6, 8.8 ± 0.8, and 6.7 ± 0.8 μM, respectively. (g) TF3 inhibited 3CL^pro^ with IC_50_ of 8.5 ± 0.7 μM based on the inhibitor concentration-dependent curve. (h) TF3 inhibited three activities of PL^pro^ with IC_50_ values of 4.7 ± 0.5, 5.5 ± 0.6, and 6.3 ± 0.8 μM, respectively, based on the inhibitor concentration-dependent curves shown in left, middle, and right panels, respectively. Proanthocyanidin inhibited (i) 3CL^pro^ (IC_50_ = 18.4 ± 1.1 μM) and (j) three activities of PL^pro^ with IC_50_ values of 2.4 ± 0.3, 3.3 ± 0.4, and 7.3 ± 0.6 μM, respectively, based on the inhibitor concentration-dependent curves shown in left, middle, and right panels, respectively. (k) Theaflavin inhibited RdRp with an IC_50_ of 40.2 ± 2.3 μM based on the inhibitor concentration-dependent curve. (l) Theaflavin-3′-gallate inhibited RdRp with an IC_50_ of 23.2 ± 0.4 μM, based on the inhibitor concentration-dependent curve. (m) TF3 inhibited RdRp with an IC_50_ of 2.3 ± 0.6 μM, based on the inhibitor concentration-dependent curve. (n) Proanthocyanidin inhibited RdRp with an IC_50_ of 23.4 ± 1.3 μM, based on the inhibitor concentration-dependent curve. All measurements were performed in triplicate to yield the averaged IC_50_ values and the standard deviations.

Using our established assays for the virus-encoded proteases 3CL^pro^ and PL^pro^,^37^ we subsequently examined the inhibitory activities of the natural products against these proteases. Catechin demonstrated no inhibition against 3CL^pro^ but inhibited PL^pro^ with IC_50_ values of 18.7 ± 3.5, 24.6 ± 3.3, and 17.8 ± 2.2 μM, with respect to the three substrates, respectively (Figure 4a left, middle, and right panels, respectively). Theaflavin inhibited 3CL^pro^ with an IC_50_ of 16.1 ± 2.5 μM (Figure 4b) and all three activities of PL^pro^ with IC_50_ values of 9.9 ± 0.9, 10.6 ± 0.9, and 11.2 ± 1.3 μM, respectively (Figure 4c left, middle, and right panels, respectively). Epitheaflagallin-3-*O*-gallate, theaflavin-3-gallate, and theaflavin-3′-gallate inhibited 3CL^pro^ specifically with IC_50_ values of 31.2 ± 2.6, 8.8 ± 0.8, and 6.7 ± 0.8 μM, respectively (Figure 4d–f). TF3 showed potent inhibition against 3CL^pro^ (IC_50_ = 8.5 ± 0.7 μM) (Figure 4g) and three activities of PL^pro^ with IC_50_ values of 4.7 ± 0.5, 5.5 ± 0.6, and 6.3 ± 0.8 μM, respectively (Figure 4h left, middle, and right panels, respectively). Proanthocyanidin not only inhibited 3CL^pro^ (IC_50_ = 18.4 ± 1.1 μM) (Figure 4i), with proteolytic activity against PL^pro^ as shown previously,^37^ but also inhibited the other two activities of PL^pro^, with IC_50_ values of 2.4 ± 0.3, 3.3 ± 0.4, and 7.3 ± 0.6 μM, respectively (Figure 4j left, middle, and right panels, respectively) against the three activities of PL^pro^. These data are summarized in Table 1.

### 3.4 Assay of Compounds for Inhibiting RdRp

We proceeded to evaluate the inhibitory activities of all of the natural products against RdRp using a commercial assay kit and our prepared RdRp complex.^42^ We found that theaflavin, theaflavin-3′-gallate, TF3, and proanthocyanidin exhibited inhibition toward RdRp with IC_50_ values of 40.2 ± 2.3, 23.2 ± 0.4, 2.3 ± 0.6, and 23.4 ± 1.3 μM, respectively (Figure 4k–n). Consistent with the computer prediction,^43^ TF3 emerged as the most effective inhibitor among them. The data are summarized in Table 1.

### 3.5 Antiviral Activities and Cytotoxicity of Natural Products

**Figure 5:**
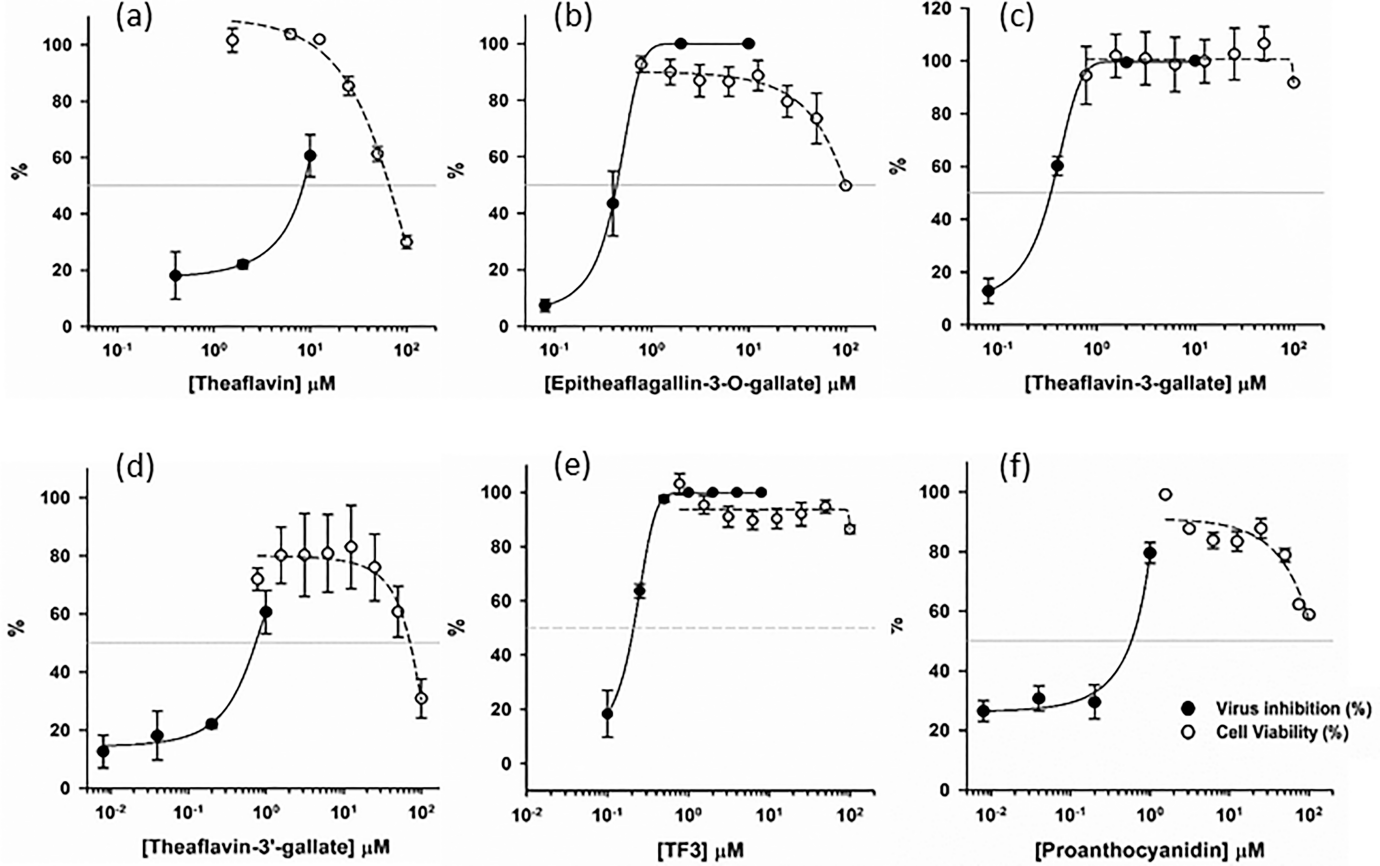
EC_50_ and CC_50_ measurements of active antivirals. (a–f) Inhibitor dose-dependent virus inhibition curves for measurements of EC_50_ values of the inhibitors theaflavin, epitheaflagallin-3-*O*-gallate, theaflavin-3-gallate, theaflavin-3′-gallate, TF3, and proanthocyanidin against the Delta variant of SARS-CoV-2 infecting Vero E6 cells are 7.4 ± 0.1, 0.5 ± 0.1, 0.4 ± 0.0, 0.8 ± 0.1, 0.2 ± 0.0, and 0.5 ± 0.1 μM, respectively. These curves were generated according to the plaque reduction data. Their CC_50_ values derived from the plots were 66.4 ± 3.2, 109.2 ± 45.3, >100, 69.9 ± 12.9, >100, and >100 μM, respectively. All of the measurements were performed in triplicate to yield the averaged EC_50_ and CC_50_ values and standard deviations.

In the final step, the natural products were subjected to the antiviral plaque reduction assay using Delta (NTU92) and Omicron BA.5 (NTU280) strains of SARS-CoV-2. To discern the stage at which the active compounds exerted their antiviral activities, these compounds were preincubated with the virus or added during infection and removed after the infection (entry treatment) or added only after infection (postentry treatment). Notably, catechin did not inhibit the Delta SARS-CoV-2 at 10 μM (figure not shown). The EC_50_ values for theaflavin, epitheaflagallin-3-*O*-gallate, theaflavin-3-gallate, theaflavin-3′-gallate, TF3, and proanthocyanidin against the entry of the Delta variant of SARS-CoV-2 into Vero E6 cells were measured to be 7.4 ± 0.1, 0.5 ± 0.1, 0.4 ± 0.0, 0.8 ± 0.1, 0.2 ± 0.0, and 0.5 ± 0.1 μM, respectively (Figure 5a–f), and the data are summarized in Table 1. The active antivirals exhibited inhibition not only against the Delta strain but also antagonized the Omicron BA.5 strain (see Figure S1 for the dose-dependent inhibition curves and Table 1 for their measured EC_50_ values). Specifically, TF3, as an example, not only inhibited Delta and Omicron BA.5 but also inhibited the wild-type, α, and γ strains with similar EC_50_ values of 0.3 ± 0.0, 0.5 ± 0.0, and 0.4 ± 0.2, respectively (see Figure S2). All of the antivirals were found to be active when added at the entry stage but not at the postentry stage, suggesting that they likely prevent virus entry to host cells rather than virus replication inside the cells. The CC_50_ values of theaflavin, epitheaflagallin-3-*O*-gallate, theaflavin-3-gallate, theaflavin-3′-gallate, TF3, and proanthocyanidin derived from the plots were 66.4 ± 3.2, 109.2 ± 45.3, >100, 69.9 ± 12.9, >100, and >100 μM, respectively, as shown in Figure 5a–f. The derived selectivity index values for these compounds were 9.0, 232.3, >285.7, 93.2, >476.2, and >188.7, respectively.

### 3.6 Computer Modeling of the Inhibitors for the Human Proteases Involved in the Virus Entry

**Figure 6:**
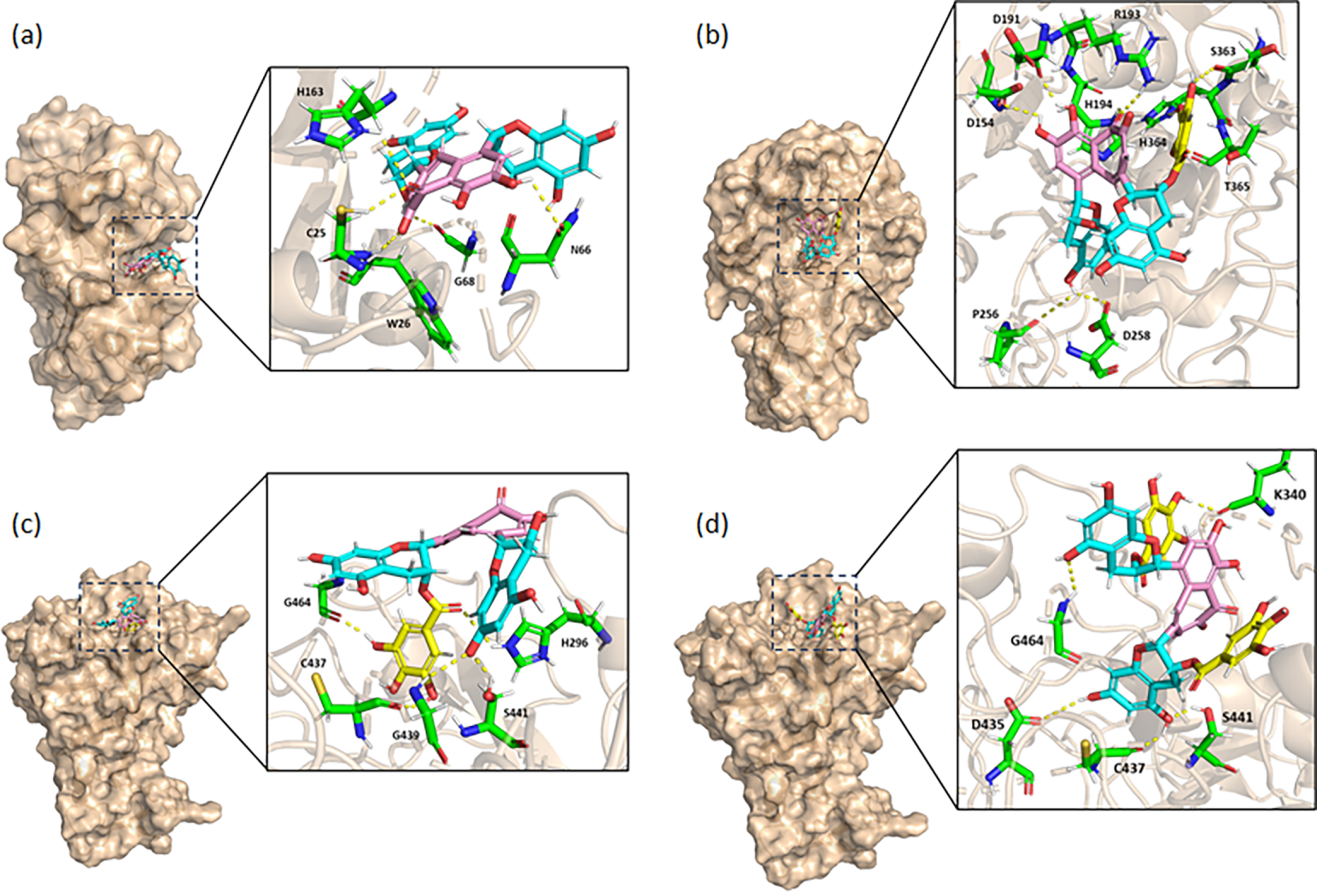
Modeled binding modes of selected natural products with cathepsin L, furin, and TMPRSS2 to rationalize their inhibition specificities. The binding modes of (a) theaflavin with cathepsin L (PDB: 5MQY), and (b) theaflavin-3-gallate with furin (PDB: 4RYD), and (c) theaflavin-3′-gallate and (d) TF3 (bottom) with TMPRSS2 (PDB: 7MEQ), respectively. These compounds represent the best inhibitors of their targets in this study. The central 3,4,6-trihydroxy-5H-benzo[7]annulen-5-one moieties are colored pink, chromane rings are in cyan, and gallate groups are shown in yellow. Oxygen atoms are shown in red. In amino acids, carbon skeletons are colored green, nitrogen atoms in blue, and sulfur atoms in yellow.

Given that most of the tea polyphenols inhibit viral entry, understanding their binding modes with key human proteases such as cathepsin L, furin, and TMPRSS2 as well as the RBD/ACE2 interaction is crucial. Based on the IC_50_ data, it appears that the smaller theaflavin inhibited only cathepsin L, while the larger epitheaflagallin-3-*O*-gallate, theaflavin-3-gallate, and theaflavin-3′-gallate inhibited furin and TMPRSS2, with the exception of theaflavin-3′-gallate also weakly inhibiting cathepsin L. However, only theaflavin-3′-gallate and even larger TF3 inhibited TMPRSS2 potently. To rationalize the structure–activity relationship, computer docking studies were conducted to explore the binding interaction of these potent inhibitors with their respective targets. In the docking analysis, theaflavin, which exhibits an IC_50_ of 11.1 μM forms several hydrogen bonds with residues C25, W26, N66, G68, and H163 of cathepsin L. Especially, the central 3,4,6-trihydroxy-5*H*-benzo[7]annulen-5-one group, highlighted in pink, forms hydrogen bonds with both catalytic residues C25 and H163, and the residue W26 of cathepsin L (Figure 6a). The two chromane rings highlighted in cyan form hydrogen bonds with residues N66 and G68. As shown in Figure 6b, theaflavin-3-gallate, the most potent inhibitor of furin in this study with an IC_50_ of 36.7 μM, forms hydrogen bonds with residues D154, D191, R193, P256, D258, S363, H364, and T365 of furin, surrounding the active site residues D153, H194, N295, and S368. Its central 3,4,6-trihydroxy-5*H*-benzo[7]annulen-5-one group, identical to that in theaflavin and depicted in pink, forms a hydrogen bond with the catalytic residue H194. An OH group on the chromane ring depicted in cyan forms hydrogen bonds with the backbone oxygen of P256 and the side-chain carboxylate oxygen of D258. Additionally, two OH groups on the gallate moiety shown in yellow form hydrogen bonds with the side-chain oxygens of S363 and T365, while one OH forms a hydrogen bond with the side-chain nitrogen of H364. On the other hand, theaflavin-3′-gallate (IC_50_ = 6.2 μM) and TF3 (IC_50_ = 5.5 μM) exhibited potent inhibition against TMPRSS2. The gallate group of theaflavin-3′-gallate, shown in yellow, occupies the catalytic pocket of TMPRSS2 and forms hydrogen bonds with residues C437, G464, and H296 around the catalytic residues H296, D435, and S441 (Figure 6c). Its chromane ring, shown in cyan, could also form hydrogen bonds with the G439 and S441 residues. TF3 also forms hydrogen bonds with residues D435, C437, and S441 of TMPRSS2 through its chromane group shown in cyan (Figure 6d). Due to the extra gallate in TF3, its binding orientation is different from that of theaflavin-3′-gallate.

## 4 Discussion

While numerous herbs and natural products have demonstrated inhibition of CoVs,^26−33^ their antiviral mechanisms remain incompletely understood. For instance, pseudotyped virus assays have facilitated the screening of natural products as SARS-CoV-2 entry inhibitors,^44^ but the precise targets, such as RBD/ACE2, TMPRSS2, furin, and/or cathepsin L, have yet to be fully identified. In this study, we chose a series of tea polyphenols with similar building blocks and structural features to investigate their exact antiviral targets. As illustrated in Figure 1 from catechin to proanthocyanidin, we identified theaflavin, epitheaflagallin-3-*O*-gallate, theaflavin-3-gallate, theaflavin-3′-gallate, TF3, and proanthocyanidin as active antivirals against SARS-CoV-2. These compounds exhibited antiviral activity only when they were preincubated with the virus and included during virus infection of cells (entry treatment) but not when added after virus infection (postentry treatment). While these compounds exhibit inhibitory activities on various targets (Table 1), their true antiviral targets are likely to be limited to those for virus entry, such as RBD/ACE2 interaction, TMPRSS2, furin, and/or cathepsin L. This inference is supported by the fact that their antiviral activities were observed specifically under entry treatment conditions.

Catechin is a flavanol characterized by a hydroxyl group at the C-3 position of the C ring and was initially regarded as a potential antiviral targeting various components of SARS-CoV-2, including proteases, RdRp, spike, and ACE2, as suggested by computer modeling.^45^ However, our current study reveals that catechin exhibits only weak inhibitory activity against PL^pro^ (Table 1), consistent with its failure to inhibit SARS-CoV-2. In contrast, theaflavin that looks like containing two molecules of catechin but with a different 7-membered ring to fuse with the benzene ring of a catechin inhibits not only the RBD/ACE2 interaction (IC_50_ = 12.3 μM) and cathepsin L activity (IC_50_ = 11.1 μM) but also activities of 3CL^pro^ (IC_50_ = 16.1 μM), PL^pro^ (IC_50_ = 9.9, 10.6, and 11.2 μM), and RdRp (IC_50_ = 40.2 μM). However, its inhibitory effect on SARS-CoV-2 with an EC_50_ of 7.4 μM against the Delta strain was only observed with entry treatment (Figure 5a), suggesting that its antiviral activity is primarily attributed to inhibiting RBD/ACE2 and cathepsin L. With an additional gallate group, both epitheaflagallin-3-*O*-gallate and theaflavin-3-gallate inhibit furin (IC_50_ = 55.0 and 36.7 μM, respectively) and TMPRSS2 (IC_50_ = 35.3 and 48.7 μM, respectively). On the other hand, theaflavin-3′-gallate inhibits all three human proteases, cathepsin L (IC_50_ = 51.1 μM), furin (IC_50_ = 58.5 μM), and TMPRSS2 more potently (IC_50_ = 6.2 μM). All three tea polyphenols exhibit approximately 10-fold more potent antiviral effect (EC_50_ = 0.5, 0.4, and 0.8 μM, respectively, against the Delta strain) than that of theaflavin. Although theaflavin-3′-gallate also inhibits 3CL^pro^ (IC_50_ = 6.7 μM) and RdRp (IC_50_ = 23.2 μM), these activities do not fully explain the antiviral effect. With two gallate groups, TF3 inhibited TMPRSS2 (IC_50_ = 5.5 μM) and RBD/ACE2 (IC_50_ = 8.7 μM), in addition to inhibiting 3CL^pro^ (IC_50_ = 8.5 μM), PL^pro^ (4.7–6.3 μM), and RdRp (IC_50_ = 2.3 μM). TF3 is demonstrated to be an effective antiviral with an EC_50_ of 0.2 μM under entry treatment by blocking the virus entry. Therefore, inhibiting RBD/ACE2 and the human protease TMPRSS2 indeed contributes to their antiviral effect, although they also inhibit 3CL^pro^, PL^pro^, and RdRp.

It is intriguing that theaflavin, smaller than TF3 and lacking two 3,4,5-hydroxybenzoyl groups at the ends (see Figure 1 for the structures), inhibits cathepsin L rather than TMPRSS2, although both compounds inhibit RBD/ACE2 to block the virus entry. In contrast, theaflavin-3′-gallate inhibits TMPRSS2 more potently (IC_50_ = 6.2 μM), although it also weakly inhibits furin and cathepsin L. Comparing the docking results of theaflavin and theaflavin-3′-gallate, it was found that a portion of the latter compound was located outside the active site cleft of cathepsin L, likely due to the presence of an additional gallate group (figure not shown). The largest TF3 with extra gallate groups inhibits only TMPRSS2, but not furin and cathepsin L. Therefore, the computer models shown in Figure 6 successfully explain the structure–activity relationship of these structurally related natural products, with the smaller theaflavin inhibiting cathepsin L and larger theaflavin-3′-gallate and TF3 inhibiting TMPRSS2.

Theaflavin, derived from black tea, has been reported to inhibit SARS-CoV-2 3CL^pro^.^46^ Additionally, it has been found to inhibit the RBD/ACE2 interaction and consequently block the pseudovirus entry.^47^ Here, we found that theaflavin also inhibits cathepsin L, further contributing to its ability to block SARS-CoV-2 entry by interfering with the RBD/ACE2 interaction. Moreover, TF3 was reported to effectively inhibiting the interaction between recombinant ACE2 and RBD at 60 μM, which is comparable to the concentration of this compound in tea beverages.^48^ TF3 is a black tea polyphenol produced by the polymerization and oxidation of green tea polyphenols epicatechin gallate and (−)-epigallocatechin-3-gallate, an antioxidant commonly found in green and black tea, during fermentation of fresh tea leaves. Previous studies have demonstrated TF3′s inhibitory effect on SARS-CoV 3CL^pro^ in vitro^29^ as well as blocking the RBD/ACE interaction.^47^ Moreover, TF3 has been shown to antagonize Zika virus’s protease activity and virus replication.^49^ In this study, we demonstrate that TF3 not only antagonizes the RBD/ACE2 (IC_50_ = 8.7 μM), but also inhibits TMPRSS2 (IC_50_ = 5.5 μM), thereby effectively blocking SARS-CoV-2 entry. The dual-inhibitory action results in a potent antiviral EC_50_ of 0.2 μM against the Delta SARS-CoV-2, without toxicity at a concentration of up to 100 μM (selectivity index SI >476.2).

Compared to the potentially repurposed drugs for COVID-19, certain herbs and natural products demonstrate antiviral properties that surpass some conventional drugs while exhibiting low toxicity.^50^ It appears that multiple targeting is a common feature of natural products with broad biological activities, including polyphenols described in this study. The antiviral ability of polyphenols may stem from the presence of multiple OH groups for forming H-bonding interactions and aromatic rings for hydrophobic and π–π interactions to inhibit the targets essential for SARS-CoV-2 entry, replication, and/or immune escaping. Moreover, their negatively charged phenolates may act like the warheads of tetrazolates as angiotensin receptor blockers (ARBs) to block RBD/ACE2, furin, and/or TMPRSS2 by complexing with the positive arginines at arginine-rich multibasic sites S1/S2 (680–686) and S2 (810–816), which catalyze the cleavage of spike protein initiating infection.^51−54^ Anionic polyphenols could act as ARBs, similar to anionic tetrazolates of antihypertensive drugs, such as elmisartan, candesartan, and losartan, which have been demonstrated to clinically protect hypertensive patients infected by COVID-19.^55^ With multiple targets, it is important to avoid misinterpretation of the antiviral mechanisms of polyphenols on the basis of only limited assays. We propose that for compounds acting as the SARS-CoV-2 entry inhibitors (effective via entry treatment), investigations should focus on accessing their impacts on targets such as RBD/ACE2, TMPRSS2, furin, and cathepsin L. Conversely, for compounds inhibiting SARS-CoV-2 replication within cells (effective via postentry treatment), assessments should include targets such as 3CL^pro^, PL^pro^, and RdRp to gain a comprehensive understanding of their true antiviral mechanisms.

## 5 Conclusions

The systematic assays conducted in the current study have provided an insight into the anti-SARS-CoV-2 mechanisms of polyphenols. Through multiple targeting, natural products related to tea polypehenols inhibit various targets, albeit not always with high potency individually. However, the combination of these inhibitory activities results in sub-μM EC_50_, demonstrating their potential effectiveness against SARS-CoV-2. Extensive cohort studies and human intervention trials on COVID-19 patients, remarking on the possibility of decreasing virus multiplication and thus improving clinical signs, could be conducted following comprehensive in vivo investigations. In fact, a study demonstrated that epigallocatechin gallate, a green tea polyphenol, reduced SARS-CoV-2 replication in a mouse model.^56^ This suggests that the incorporation of polyphenols into food and clinical practice should be swiftly implemented, as they are natural mixtures derived from plants, already approved for use as herbs and food. Importantly, the use of polyphenols offers the advantage of a high safety profile with minimal risk of causing major side effects. Therefore, our studies presented here not only elucidate the antiviral mechanisms of certain natural products but also offer effective options for preventing and/or treating COVID-19 with these anti-SARS-CoV-2 natural products.

## Supporting Information Available

EC_50_ profiles of natural products against Omicron BA.5 and that of TF3 against different strains of SARS-CoV-2 (PDF)

## Abbreviations

3CL^pro^: 3C-like protease
ACE2: angiotensin-converting enzyme 2
ACP: acid phosphatase
ARBs: angiotensin receptor blockers
CoV: coronavirus
COVID-19: coronavirus disease 2019
DTT: dithiothreitol
DMEM: Dulbecco’s modified Eagle medium
FBS: fetal bovine serum
FDA: food and drug administration
MERS: Middle East respiratory syndrome
NSP: nonstructural protein
PBS: phosphate-buffered saline
pfu: plaque-forming units
PL^pro^: papain-like protease
RdRp: RNA-dependent RNA polymerase
SARS: severe acute respiratory syndrome
TF3: theaflavin-3,3′-digallate
TMPRSS2: transmembrane protease serine 2
WHO: World Health Organization

## References

[ref1] Zhu N.; Zhang D.; Wang W.; Li X.; Yang B.; Song J.; Zhao X.; Huang B.; Shi W.; Lu R.; Niu P.; Zhan F.; Ma X.; Wang D.; Xu W.; Wu G.; Gao G. F.; Tan W. China novel coronavirus investigating and research team. A novel coronavirus from patients with pneumonia in China, 2019. N. Engl. J. Med. 2020, 382 (8), 727–733. 10.1056/NEJMoa2001017. 31978945 PMC7092803

[ref2] Huang C.; Wang Y.; Li X.; Ren L.; Zhao J.; Hu Y.; Zhang L.; Fan G.; Xu J.; Gu X.; Cheng Z.; Yu T.; Xia J.; Wei Y.; Wu W.; Xie X.; Yin W.; Li H.; Liu M.; Xiao Y.; Gao H.; Guo L.; Xie J.; Wang G.; Jiang R.; Gao Z.; Jin Q.; Wang J.; Cao B. Clinical features of patients infected with 2019 novel coronavirus in Wuhan, China. Lancet 2020, 395 (10223), 497–506. 10.1016/S0140-6736(20)30183-5. 31986264 PMC7159299

[ref3] Peiris J. S.; Lai S. T.; Poon L. L.; Guan Y.; Yam L. Y.; Lim W.; Nicholls J.; Yee W. K.; Yan W. W.; Cheung M. T.; Cheng V. C.; Chan K. H.; Tsang D. N.; Yung R. W.; Ng T. K.; Yuen K. Y.; Coronavirus as a possible cause of severe acute respiratory syndrome. Lancet 2003, 361 (9366), 1319–1325. 10.1016/s0140-6736(03)13077-2. 12711465 PMC7112372

[ref4] Ksiazek T. G.; Erdman D.; Goldsmith C. S.; Zaki S. R.; Peret T.; Emery S.; Tong S.; Urbani C.; Comer J. A.; Lim W.; Rollin P. E.; Dowell S. F.; Ling A. E.; Humphrey C. D.; Shieh W. J.; Guarner J.; Paddock C. D.; Rota P.; Fields B.; DeRisi J.; Yang J. Y.; Cox N.; Hughes J. M.; LeDuc J. W.; Bellini W. J.; Anderson L. J.; A novel coronavirus associated with severe acute respiratory syndrome. N. Engl. J. Med. 2003, 348 (20), 1953–1966. 10.1056/NEJMoa030781. 12690092

[ref5] Hoffmann M.; Kleine-Weber H.; Schroeder S.; Krüger N.; Herrler T.; Erichsen S.; Schiergens T. S.; Herrler G.; Wu N. H.; Nitsche A.; Müller M. A.; Drosten C.; Pöhlmann S. SARS-CoV-2 cell entry depends on ACE2 and TMPRSS2 and is blocked by a clinically proven protease inhibitor. Cell 2020, 181 (2), 271–280.e8. 10.1016/j.cell.2020.02.052. 32142651 PMC7102627

[ref6] Zhao M. M.; Yang W. L.; Yang F. Y.; Zhang L.; Huang W. J.; Hou W.; Fan C. F.; Jin R. H.; Feng Y. M.; Wang Y. C.; Yang J. K. Cathepsin L plays a key role in SARS-CoV-2 infection in humans and humanized mice and is a promising target for new drug development. Signal Transduction Targeted Ther. 2021, 6 (1), 134 10.1038/s41392-021-00558-8. PMC7997800 33774649

[ref7] Wu F.; Zhao S.; Yu B.; Chen Y. M.; Wang W.; Song Z. G.; Hu Y.; Tao Z. W.; Tian J. H.; Pei Y. Y.; Yuan M. L.; Zhang Y. L.; Dai F. H.; Liu Y.; Wang Q. M.; Zheng J. J.; Xu L.; Holmes E. C.; Zhang Y. Z. A new coronavirus associated with human respiratory disease in China. Nature 2020, 579 (7798), 265–269. 10.1038/s41586-020-2008-3. 32015508 PMC7094943

[ref8] Lei J.; Kusov Y.; Hilgenfeld R. Nsp3 of coronaviruses: Structures and functions of a large multi-domain protein. Antiviral Res. 2018, 149, 58–74. 10.1016/j.antiviral.2017.11.001. 29128390 PMC7113668

[ref9] Freitas B. T.; Durie I. A.; Murray J.; Longo J. E.; Miller H. C.; Crich D.; Hogan R. J.; Tripp R. A.; Pegan S. D. Characterization and noncovalent inhibition of the deubiquitinase and deISGylase activity of SARS-CoV-2 papain-like protease. ACS Infect. Dis. 2020, 6 (8), 2099–2109. 10.1021/acsinfecdis.0c00168. 32428392

[ref10] Kirchdoerfer R. N.; Ward A. B. Structure of the SARS-CoV nsp12 polymerase bound to nsp7 and nsp8 co-factors. Nat. Commun. 2019, 10 (1), 2342 10.1038/s41467-019-10280-3. 31138817 PMC6538669

[ref11] Subissi L.; Posthuma C. C.; Collet A.; Zevenhoven-Dobbe J. C.; Gorbalenya A. E.; Decroly E.; Snijder E. J.; Canard B.; Imbert I. One severe acute respiratory syndrome coronavirus protein complex integrates processive RNA polymerase and exonuclease activities. Proc. Natl. Acad. Sci. U.S.A. 2014, 111 (37), E3900–E3909. 10.1073/pnas.1323705111. 25197083 PMC4169972

[ref12] Ivanov K. A.; Thiel V.; Dobbe J. C.; van der Meer Y.; Snijder E. J.; Ziebuhr J. Multiple enzymatic activities associated with severe acute respiratory syndrome coronavirus helicase. J. Virol. 2004, 78 (11), 5619–5632. 10.1128/JVI.78.11.5619-5632.2004. 15140959 PMC415832

[ref13] Bouvet M.; Imbert I.; Subissi L.; Gluais L.; Canard B.; Decroly E. RNA 3′-end mismatch excision by the severe acute respiratory syndrome oronavirus nonstructural protein nsp10/nsp14 exoribonuclease complex. Proc. Natl. Acad. Sci. U.S.A. 2012, 109 (24), 9372–9377. 10.1073/pnas.1201130109. 22635272 PMC3386072

[ref14] Tao K.; Tzou P. L.; Nouhin J.; Bonilla H.; Jagannathan P.; Shafer R. W. SARS-CoV-2 antiviral therapy. Clin. Microbiol. Rev. 2021, 34 (4), e0010921 10.1128/CMR.00109-21. 34319150 PMC8404831

[ref15] Tan H.; Hu Y.; Jadhav P.; Tan B.; Wang J. Progress and challenges in targeting the SARS-CoV-2 papain-like protease. J. Med. Chem. 2022, 65 (11), 7561–7580. 10.1021/acs.jmedchem.2c00303. 35620927 PMC9159073

[ref16] Li G.; Hilgenfeld R.; Whitley R.; De Clercq E. Therapeutic strategies for COVID-19: Progress and lessons learned. Nat. Rev. Drug Discovery 2023, 22 (6), 449–475. 10.1038/s41573-023-00672-y. 37076602 PMC10113999

[ref17] Takashita E.; Yamayoshi S.; Simon V.; van Bakel H.; Sordillo E. M.; Pekosz A.; Fukushi S.; Suzuki T.; Maeda K.; Halfmann P.; Sakai-Tagawa Y.; Ito M.; Watanabe S.; Imai M.; Hasegawa H.; Kawaoka Y. Efficacy of Antibodies and Antiviral Drugs against Omicron BA.2.12.1, BA.4, and BA.5 Subvariants. N. Engl. J. Med. 2022, 387 (5), 468–470. 10.1056/NEJMc2207519. 35857646 PMC9342381

[ref18] Gordon C. J.; Tchesnokov E. P.; Woolner E.; Perry J. K.; Feng J. Y.; Porter D. P.; Götte M. Remdesivir is a direct-acting antiviral that inhibits RNA-dependent RNA polymerase from Severe Acute Respiratory Syndrome Coronavirus 2 with high potency. J. Biol. Chem. 2020, 295 (20), 6785–6797. 10.1074/jbc.RA120.013679. 32284326 PMC7242698

[ref19] Grein J.; Ohmagari N.; Shin D.; Diaz G.; Asperges E.; Castagna A.; Feldt T.; Green G.; Green M. L.; Lescure F. X.; Nicastri E.; Oda R.; Yo K.; Quiros-Roldan E.; Studemeister A.; Redinski J.; Ahmed S.; Bernett J.; Chelliah D.; Chen D.; Chihara S.; Cohen S. H.; Cunningham J.; D’Arminio Monforte A.; Ismail S.; Kato H.; Lapadula G.; L’Her E.; Maeno T.; Majumder S.; Massari M.; Mora-Rillo M.; Mutoh Y.; Nguyen D.; Verweij E.; Zoufaly A.; Osinusi A. O.; DeZure A.; Zhao Y.; Zhong L.; Chokkalingam A.; Elboudwarej E.; Telep L.; Timbs L.; Henne I.; Sellers S.; Cao H.; Tan S. K.; Winterbourne L.; Desai P.; Mera R.; Gaggar A.; Myers R. P.; Brainard D. M.; Childs R.; Flanigan T. Compassionate use of Remdesivir for patients with severe Covid-19. N. Engl. J. Med. 2020, 382 (24), 2327–2336. 10.1056/NEJMoa2007016. 32275812 PMC7169476

[ref20] Bernal A. J.; da Silva M. M. G.; Musungaie D. B.; Kovalchuk E.; Gonzalez A.; Reyes V. D.; Martín-Quirós A.; Caraco Y.; Williams-Diaz A.; Brown M. L.; Du J.; Pedley A.; Assaid C.; Strizki J.; Grobler J. A.; Shamsuddin H. H.; Tipping R.; Wan H.; Paschke A.; Butterton J. R.; Johnson M. G.; De Anda C.; Molnupiravir for oral treatment of Covid-19 in nonhospitalized patients. N. Engl. J. Med. 2022, 386 (6), 509–520. 10.1056/NEJMoa2116044. 34914868 PMC8693688

[ref21] Hammond J.; Leister-Tebbe H.; Gardner A.; Abreu P.; Bao W.; Wisemandle W.; Baniecki M. L.; Hendrick V. M.; Damle B.; Simón-Campos A.; Pypstra R.; Rusnak J. M.; Oral Nirmatrelvir for high-risk, nonhospitalized adults with Covid-19. N. Engl. J. Med. 2022, 386 (15), 1397–1408. 10.1056/NEJMoa2118542. 35172054 PMC8908851

[ref22] Gandhi S.; Klein J.; Robertson A. J.; Peña-Hernández M. A.; Lin M. J.; Roychoudhury P.; Lu P.; Fournier J.; Ferguson D.; Bakhash S. A. K. M.; Muenker M. C.; Srivathsan A.; Wunder E. A. Jr; Kerantzas N.; Wang W.; Lindenbach B.; Pyle A.; Wilen C. B.; Ogbuagu O.; Greninger A. L.; Iwasaki A.; Schulz W. L.; Ko A. I. De novo emergence of a remdesivir resistance mutation during treatment of persistent SARS-CoV-2 infection in an immunocompromised patient: a case report. Nat. Commun. 2022, 13 (1), 1547 10.1038/s41467-022-29104-y. 35301314 PMC8930970

[ref23] Heyer A.; Günther T.; Robitaille A.; Lütgehetmann M.; Addo M. M.; Jarczak D.; Kluge S.; Aepfelbacher M.; zur Wiesch J. S.; Fischer N.; Grundhoff A. Remdesivir-induced emergence of SARS-CoV2 variants in patients with prolonged infection. Cell Rep. Med. 2022, 3 (9), 100735 10.1016/j.xcrm.2022.100735. 36075217 PMC9378267

[ref24] Ip J. D.; Wing-Ho Chu A.; Chan W. M.; Cheuk-Ying Leung R.; Abdullah S. M. U.; Sun Y.; Kai-Wang To K. Global prevalence of SARS-CoV-2 3CL protease mutations associated with nirmatrelvir or ensitrelvir resistance. EBioMedicine 2023, 91, 104559 10.1016/j.ebiom.2023.104559. 37060743 PMC10101811

[ref25] Hu Y.; Lewandowski E.; Tan H.; Zhang X.; Morgan R. T.; Zhang X.; Jacobs L. M. C.; Butler S. G.; Gongora M. V.; Choy J.; Deng X.; Chen Y.; Wang J. Naturally occurring mutations of SARS-CoV-2 main protease confer drug resistance to nirmatrelvir. ACS Cent. Sci. 2023, 9 (8), 1658–1669. 10.1021/acscentsci.3c00538. 37637734 PMC10451032

[ref26] Jantan I.; Arshad L.; Septama A. W.; Haque M. A.; Mohamed-Hussein Z. A.; Govender N. T. Antiviral effects of phytochemicals against severe acute respiratory syndrome coronavirus 2 and their mechanisms of action: A review. Phytother. Res. 2023, 37 (3), 1036–1056. 10.1002/ptr.7671. 36343627 PMC9878073

[ref27] Wu C. Y.; Jan J. T.; Ma S. H.; Kuo C. J.; Juan H. F.; Cheng Y. S.; Hsu H. H.; Huang H. C.; Wu D.; Brik A.; Liang F. S.; Liu R. S.; Fang J. M.; Chen S. T.; Liang P. H.; Wong C. H. Small molecules targeting severe acute respiratory syndrome human coronavirus. Proc. Natl. Acad. Sci. U.S.A. 2004, 101 (27), 10012–10017. 10.1073/pnas.0403596101. 15226499 PMC454157

[ref28] Wen C. C.; Kuo Y. H.; Jan J. T.; Liang P. H.; Wang S. Y.; Liu H. G.; Lee C. K.; Chang S. T.; Kuo C. J.; Lee S. S.; Hou C. C.; Hsiao P. W.; Chien S. C.; Shyur L. F.; Yang N. S. Specific plant terpenoids and lignoids possess potent antiviral activities against severe acute respiratory syndrome coronavirus. J. Med. Chem. 2007, 50 (17), 4087–4095. 10.1021/jm070295s. 17663539

[ref29] Chen C. N.; Lin C. P.; Huang K. K.; Chen W. C.; Hsieh H. P.; Liang P. H.; Hsu J. T. Inhibition of SARS-CoV 3C-like protease activity by Theaflavin-3,3′-digallate (TF3). Evidence-Based Complementary Altern. Med. 2005, 2 (2), 209–215. 10.1093/ecam/neh081. PMC1142193 15937562

[ref30] Islam M. T.; Sarkar C.; El-Kersh D. M.; Jamaddar S.; Uddin S. J.; Shilpi J. A.; Mubarak M. S. Natural products and their derivatives against coronaviruses: A review of the non-clinical and pre-clinical data. Phytother. Res. 2020, 34 (10), 2471–2492. 10.1002/ptr.6700. 32248575

[ref31] Llivisaca-Contreras S. A.; Naranjo-Morán J.; Pino-Acosta A.; Pieters L.; Berghe W. V.; Manzano P.; Vargas-Pérez J.; León-Tamariz F.; Cevallos-Cevallos J. M. Plants and natural products with activity against various types of coronaviruses: A review with focus on SARS-CoV-2. Molecules 2021, 26 (13), 4099 10.3390/molecules26134099. 34279439 PMC8271932

[ref32] Chapman R. L.; Andurkar S. V. A review of natural products, their effects on SARS-CoV-2 and their utility as lead compounds in the discovery of drugs for the treatment of COVID-19. Med. Chem. Res. 2022, 31 (1), 40–51. 10.1007/s00044-021-02826-2. 34873386 PMC8636070

[ref33] Zhao Y.; Deng S.; Bai Y.; Guo J.; Kai G.; Huang X.; Jia X. Promising natural products against SARS-CoV-2: Structure, function, and clinical trials. Phytother. Res. 2022, 36, 3833–3858. 10.1002/ptr.7580. 35932157 PMC9538226

[ref34] Fraser B. J.; Beldar S.; Seitova A.; Hutchinson A.; Mannar D.; Li Y.; Kwon D.; Tan R.; Wilson R. P.; Leopold K.; Subramaniam S.; Halabelian L.; Arrowsmith C. H.; Bénard F. Structure and activity of human TMPRSS2 protease implicated in SARS-CoV-2 activation. Nat. Chem. Biol. 2022, 18 (9), 963–971. 10.1038/s41589-022-01059-7. 35676539

[ref35] Palla S. R.; Lee C. W.; Chao T. L.; Lo H. L. V.; Liu J. J.; Pan M. Y. C.; Chiu Y. T.; Lin W. C.; Hu C. W.; Yang C. M.; Chen Y. Y.; Fang J. T.; Lin S. W.; Lin Y. T.; Lin H. C.; Kuo C. J.; Wang L. H. C.; Chang S. Y.; Liang P. H. Synthesis, evaluation, and mechanism of 1-(4-(arylethylenylcarbonyl)phenyl)-4-carboxy-2-pyrrolidinones as potent reversible SARS-CoV-2 entry inhibitors. Antiviral Res. 2023, 219, 105735 10.1016/j.antiviral.2023.105735. 37858764

[ref36] Lee R.-L.; Li T. N.; Chang S. Y.; Chao T. L.; Kuo C. H.; Pan M. Y.; Chiou Y. T.; Liao K. J.; Yang Y.; Wu Y. H.; Huang C. H.; Juan H. F.; Hsieh H. P.; Wang L. H. Identification of entry inhibitors against delta and omicron variants of SARS-CoV-2. Int. J. Mol. Sci. 2022, 23 (7), 4050 10.3390/ijms23074050. 35409412 PMC8999638

[ref37] Kuo C. J.; Chao T. L.; Kao H. C.; Tsai Y. M.; Liu Y. K.; Wang L. H.; Hsieh M. C.; Chang S. Y.; Liang P. H. Kinetic characterization and inhibitor screening for the proteases leading to identification of drugs against SARS-CoV-2. Antimicrob. Agents Chemother. 2021, 65 (4), e02577-20 10.1128/AAC.02577-20. 33526482 PMC8097444

[ref38] Ma C.; Hu Y.; Townsend J. A.; Lagarias P. I.; Marty M. T.; Kolocouris A.; Wang J. Ebselen, Disulfiram, Carmofur, PX-12, Tideglusib, and Shikonin are nonspecific promiscuous SARS-CoV-2 main protease inhibitors. ACS Pharmacol. Transl. Sci. 2020, 3 (6), 1265–1277. 10.1021/acsptsci.0c00130. 33330841 PMC7571300

[ref39] Bertolin A. P.; Weissmann F.; Zeng J.; Posse V.; Milligan J. C.; Canal B.; Ulferts R.; Wu M.; Drury L. S.; Howell M.; et al. Identifying SARS-CoV-2 antiviral compounds by screening for small molecule inhibitors of nsp12/7/8 RNA-dependent RNA polymerase. Biochem. J. 2021, 478 (13), 2445–2464. 10.1042/BCJ20210200. 34198323 PMC8286815

[ref40] Shannon A.; Selisko B.; Le N.-T.-T.; Huchting J.; Touret F.; Piorkowski G.; Fattorini V.; Ferron F.; Decroly E.; Meier C.; Coutard B.; Peersen O.; Canard B. Rapid incorporation of Favipiravir by the fast and permissive viral RNA polymerase complex results in SARS-CoV-2 lethal mutagenesis. Nat. Commun. 2020, 11 (1), 4682 10.1038/s41467-020-18463-z. 32943628 PMC7499305

[ref41] Lo H. S.; Hui K. P. Y.; Lai H. M.; He X.; Khan K. S.; Kaur S.; Huang J.; Li Z.; Chan A. K. N.; Cheung H. H.; Ng K. C.; Ho J. C. W.; Chen Y. W.; Ma B.; Cheung P. M.; Shin D.; Wang K.; Lee M. H.; Selisko B.; Eydoux C.; Guillemot J. C.; Canard B.; Wu K. P.; Liang P. H.; Dikic I.; Zuo Z.; Chan F. K. L.; Hui D. S. C.; Mok V. C. T.; Wong K. B.; Mok C. K. P.; Ko H.; Aik W. S.; Chan M. C. W.; Ng W. L. Simeprevir potently suppresses SARS-CoV-2 replication and synergizes with remdesivir. ACS Cent. Sci. 2021, 7 (5), 792–802. 10.1021/acscentsci.0c01186. 34075346 PMC8056950

[ref42] Tang W.-F.; Chang Y.-H.; Lin C.-C.; Jheng J.-R.; Hsieh C.-F.; Chin Y.-F.; Chang T.-Y.; Lee J.-C.; Liang P.-H.; Lin C.-Y.; Lin G.-H.; Cai J.-Y.; Chen Y.-L.; Chen Y.-S.; Tsai S.-K.; Liu P.-C.; Yang C.-M.; Shadbahr T.; Tang J.; Hsu Y.-L.; Huang C.-H.; Wang L.-Y.; Chen C. C.; Kau J.-H.; Hung Y.-J.; Lee H.-Y.; Wang W.-C.; Tsai H.-P.; Horng J.-T. BPR3P0128, a non-nucleoside RNA-dependent RNA polymerase inhibitor, inhibits SARS-CoV-2 variants of concern and exerts synergistic antiviral activity in combination with remdesivir. Antimicrob. Agents Chemother. 2024, 68 (4), e0095623 10.1128/aac.00956-23. 38446062 PMC10989008

[ref43] Lung J.; Lin Y. S.; Yang Y. H.; Chou Y. L.; Shu L. H.; Cheng Y. C.; Liu H. T.; Wu C. Y. The potential chemical structure of anti-SARS-CoV-2 RNA-dependent RNA polymerase. J. Med. Virol. 2020, 92 (6), 693–697. 10.1002/jmv.25761. 32167173 PMC7228302

[ref44] González-Maldonado P.; Alvarenga N.; Burgos-Edwards A.; Flores-Giubi M. E.; Barúa J. E.; Romero-Rodríguez M. C.; Soto-Rifo R.; Valiente-Echeverría F.; Langjahr P.; Cantero-González G.; Sotelo P. H. Screening of natural products inhibitors of SARS-CoV-2 entry. Molecules 2022, 27 (5), 1743 10.3390/molecules27051743. 35268843 PMC8911944

[ref45] Mouffouk C.; Mouffouk S.; Mouffouk S.; Hambaba L.; Haba H. Flavonols as potential antiviral drugs targeting SARS-CoV-2 proteases (3CLpro and PLpro), spike protein, RNA-dependent RNA polymerase (RdRp) and angiotensin-converting enzyme II receptor (ACE2). Eur. J. Pharmacol. 2021, 891, 173759 10.1016/j.ejphar.2020.173759. 33249077 PMC7691142

[ref46] Jang M.; Park Y. I.; Cha Y. E.; Park R.; Namkoong S.; Lee J. I.; Park J. Tea polyphenols EGCG and theaflavin inhibit the activity of SARS-CoV-2 3CL-protease in vitro. Evidence-Based Complementary Altern. Med. 2020, 2020, 5630838 10.1155/2020/5630838. PMC7499281 32963564

[ref47] Goc A.; Sumera W.; Rath M.; Niedzwiecki A. Phenolic compounds disrupt spike-mediated receptor-binding and entry of SARS-CoV-2 pseudo-virions. PLoS One 2021, 16 (6), e0253489 10.1371/journal.pone.0253489. 34138966 PMC8211150

[ref48] Ohgitani E.; Shin-Ya M.; Ichitani M.; Kobayashi M.; Takihara T.; Kawamoto M.; Kinugasa H.; Mazda O. Significant inactivation of SARS-CoV-2 in vitro by a green tea catechin, a catechin-derivative, and black tea galloylated theaflavins. Molecules 2021, 26 (12), 3572 10.3390/molecules26123572. 34208050 PMC8230566

[ref49] Cui X.; Zhou R.; Huang C.; Zhang R.; Wang J.; Zhang Y.; Ding J.; Li X.; Zhou J.; Cen S. Identification of theaflavin-3,3′-digallate as a novel Zika virus protease inhibitor. Front. Pharmacol. 2020, 11, 514313 10.3389/fphar.2020.514313. 33192499 PMC7609463

[ref50] Fuzimoto A. D.; Isidoro C. The antiviral and coronavirus-host protein pathways inhibiting properties of herbs and natural compounds-Addiitonal weapons in the fight against the COVID-19 pandemic. J. Trandit. Complementary Med. 2020, 10 (4), 405–419. 10.1016/j.jtcme.2020.05.003. PMC7260130 32691005

[ref51] Ridgway H.; Moore G. J.; Mavromoustakos T.; Tsiodras S.; Ligielli I.; Kelaidonis K.; Chasapis C. T.; Gadanec L. K.; Zulli A.; Apostolopoulos V.; Petty R.; Karakasiliotis I.; Gorgoulis V. G.; Matsoukas J. M. Discovery of a new generation of angiotensin receptor blocking drugs: Receptor mechanisms and in silico binding to enzymes relevant to SARS-CoV-2. Comput. Struct. Biotechnol. J. 2022, 20, 2091–2111. 10.1016/j.csbj.2022.04.010. 35432786 PMC8994259

[ref52] Ridgway H.; Chasapis C. T.; Kelaidonis K.; Ligielli I.; Moore G. J.; Gadanec L. K.; Zulli A.; Apostolopoulos V.; Mavromoustakos T.; Matsoukas J. M. Understanding the driving forces that trigger mutations in SARS-CoV-2: Mutational energetics and the role of arginine blockers in COVID-19 therapy. Viruses 2022, 14 (5), 1029 10.3390/v14051029. 35632769 PMC9143829

[ref53] Ridgway H.; Orbell J. D.; Matsoukas M. T.; Kelaidonis K.; Moore G. J.; Tsiodras S.; Gorgoulis V. G.; Chasapis C. T.; Apostolopoulos V.; Matsoukas J. M. W254 in furin functions as a molecular gate promoting anti-viral drug binding: Elucidation of putative drug tunneling and docking by non-equilibrium molecular dynamics. Comput. Struct. Biotechnol. J. 2023, 21, 4589–4612. 10.1016/j.csbj.2023.09.003. 37817778 PMC10561063

[ref54] Ridgway H.; Ntallis C.; Chasapis C. T.; Kelaidonis K.; Matsoukas M. T.; Plotas P.; Apostolopoulos V.; Moore G.; Tsiodras S.; Paraskevis D.; Mavromoustakos T.; Matsoukas J. M. Molecular epidemiology of SARS-CoV-2: The dominant role of arginine in mutations and infectivity. Viruses 2023, 15 (2), 309 10.3390/v15020309. 36851526 PMC9963001

[ref55] Swiderski J.; Gadanec L. K.; Apostolopoulos V.; Moore G. J.; Kelaidonis K.; Matsoukas J. M.; Zulli A. Role of angiotensin II in cardiovascular diseases: Introducing bisartans as a novel therapy for coronavirus 2019. Biomolecules 2023, 13 (5), 787 10.3390/biom13050787. 37238657 PMC10216788

[ref56] Park R.; Jang M.; Park Y. I.; Park Y.; Jung W.; Park J.; Park J. Epigallocatechin gallate (EGCG), a green tea polyphenol, reduces coronavirus replication in a mouse model. Viruses 2021, 13 (12), 2533 10.3390/v13122533. 34960802 PMC8704347

