# Supplementary material for: Systematic Studies on the Anti-SARS-CoV-2 Mechanisms of Tea Polyphenol-Related Natural Products
Source: ACS Omega. 2024 May 17;9(22):23984–97. doi: 10.1021/acsomega.4c02392 (PMC11154727; doi:10.1021/acsomega.4c02392)
Supplement: Supplementary file 1 — ao4c02392_si_001.pdf [file ao4c02392_si_001.pdf]

Systematic Studies on the Anti-SARS-CoV-2 Mechanisms of Tea Polyphenols Related Natural Products

Chen-Wei Li[b], Tai-Ling Chao[c], Chin-Lan Lai[b], Cheng-Chin Lin[b], Max Yu-Chen Pan[d], Chieh-Ling Cheng[b], Chih-Jung Kuo[e], Lily Hui-Ching Wang[d], Sui-Yuan Chang[c, f*], Po-Huang Liang[a, b*]

[a]Institute of Biological Chemistry, Academia Sinica, Taipei 11529, Taiwan

[b]Institute of Biochemical Sciences, National Taiwan University, Taipei 10617, Taiwan

[c]Department of Clinical Laboratory Sciences and Medical Biotechnology, National Taiwan University, Taipei 10048, Taiwan

[d]Institute of Molecular and Cellular Biology, National Tsing Hua University, Hsinchu 30013, Taiwan

[e]Department of Veterinary Medicine, National Chung Hsing University, Taichung 40227, Taiwan

[f]Department of Laboratory Medicine, National Taiwan University Hospital, Taipei 10002, Taiwan

*Correspondence to:

Sui-Yuan Chang, sychang@ntu.edu.tw; Phone number: +886-2-23123456 ext. 66908.

Po-Huang Liang, phliang@gate.sinica.edu.tw; Phone number: +886-2-33664069;

Fax: +886-2-23635038.

Supporting Information

Figure S1

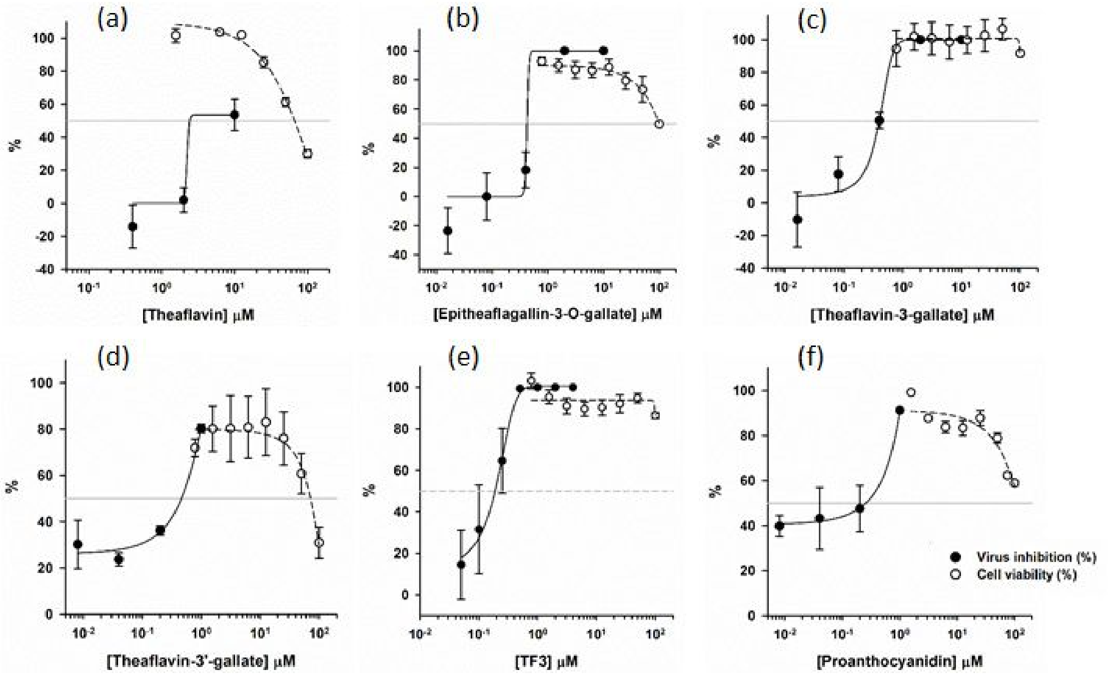

Figure S1. Antiviral activities of the natural products against Omicron BA.5 mutant strain. The cell toxicity and dose-dependent curves of the natural products, (A) Theaflavin, (B) Epitheaflagallin-3-O-gallate, (C) Theaflavin-3-gallate, (D) Theaflavin-3‘-gallate, (E) TF3, and (F) Proanthocyanidin against Omicron BA.5 mutant strain were accessed in VeroE6 cells. The EC_{50} values of Theaflavin, Epitheaflagallin-3-O-gallate, Theaflavin-3-gallate, Theaflavin-3‘-gallate, TF3, and Proanthocyanidin against the Omicron BA.5 variant of SARS-CoV-2 were measured to be 2.2 ± 0.1, 0.6 ± 0.3, 0.4 ± 0.0, 0.4 ± 0.1, 0.2 ± 0.1, and 0.2 ± 0.2 μM, respectively, based on the plaque reduction assays.

Figure S2

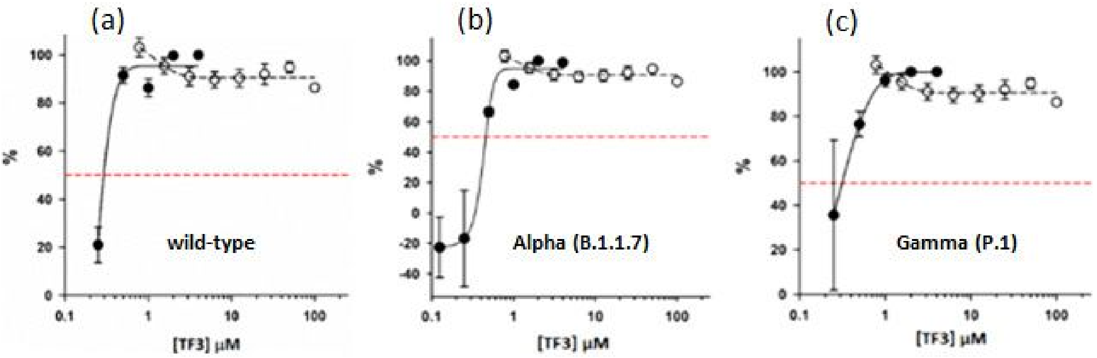

Figure S2. Efficacy and cytotoxicity of TF3 against the China wild-type, UK B.1.1.7 N501Y Alpha, and the Brazil P.1 Gamma mutant SARS-CoV-2. (A-C) The EC_{50} values of TF3 against the wild-type, Alpha, and Gamma mutant strains were measured to be 0.3 ± 0.0, 0.5 ± 0.0, and 0.4 ± 0.2 μM, respectively, based on the plaque reduction data. Its CC_{50} was >100 μM.
